# Relationship between prematurity and difficulties in the transition of food consistency in childhood: a systematic review

**DOI:** 10.1590/2317-1782/20242023100en

**Published:** 2024-05-31

**Authors:** Hellen Nataly Correia Lagos Guimarães, Renata Henrique Petreça, Sheila Tamanini de Almeida, Flavio Magno, Rosane Sampaio Santos, Karinna Veríssimo Meira Taveira, Cristiano Miranda de Araujo, Adriane Celli

**Affiliations:** 1 Programa de Pós-graduação em Saúde da Criança e do Adolescente, Universidade Federal do Paraná – UFPR - Curitiba (PR), Brasil.; 2 Departamento de Fonoaudiologia, Universidade Federal de Ciências da Saúde de Porto Alegre – UFCSPA - Porto Alegre (RS) Brasil.; 3 Programa de Distúrbios da Comunicação, Universidade Tuiuti do Paraná – UTP - Curitiba (PR), Brasil.; 4 Departamento de Morfologia, Universidade Federal do Rio Grande do Norte – UFRN - Natal, RN, Brasil.; 5 Programa de Pós-graduação em Saúde da Criança e do Adolescente, Universidade Federal do Paraná – UFPR - Curitiba (PR), Brasil.

**Keywords:** Complementary Feeding, Eating Behavior, Premature Infant, Infant, Speech Therapy

## Abstract

**Purpose:**

To systematically review the literature regarding the impact of prematurity on the transition of food consistencies in infants during the introduction of complementary feeding.

**Research strategies:**

Searches were conducted in the EMBASE, Latin American and Caribbean Literature in Health Sciences (LILACS), LIVIVO, PubMed/Medline, Scopus, and Web of Science databases, Google Scholar; for gray literature, searches were conducted on Open Gray, and ProQuest Dissertations & Theses databases, from August 10, 2020, onwards.

**Selection criteria:**

“PECOS” was selected to determine inclusion criteria: Population (P): Infants; Exposure (E): Prematurity; Comparison (C): Full-term newborns; Outcomes (O): Progression of food consistencies in premature newborns with or without comparison; Study design (S): Cohort study, Case-control; Cross-sectional.

**Data analysis:**

The methodological quality of the selected observational studies was assessed using the Meta-Analysis of Statistics Assessment and Review Instrument (MASTARI).

**Results:**

A total of 3,310 articles were found, of which nine were selected for qualitative synthesis. Among the selected studies, a relationship between invasive oral interventions and feeding difficulties was observed for all assessed skills, with feeding difficulties being more frequent in infants with lower gestational age.

**Conclusion:**

Most studies found no significant relationship between prematurity and difficulties in the progression of food consistencies during the introduction of complementary feeding; only three studies demonstrated such a relationship.

## INTRODUCTION

In Brazil, approximately three million infants are born every year, more specifically 2,849,146, of which 11% are premature^(1).^ With the advancement of technologies, the survival of this population has been increasing. However, this improved survival highlights that premature infants are deprived of crucial intrauterine brain development, resulting in consequences such as an increased frequency of comorbidities, developmental delays, and difficulties related to feeding^(2,3)^.

The introduction of complementary feeding in premature infants at six months of corrected age can improve food acceptability, as infants have more sensory experiences and are more neurologically organized. Moreover, movement patterns develop from the global motor to the fine motor; thus, understanding that the ability to eat is a fine motor skill, global motor development is essential for good oral function. Oral stability depends on head and shoulder control, which are related to trunk and pelvic stability, and thus influenced by global motor development. As motor development progresses, complex functions and movements can be performed by the child^(4-6)^.

A literature review described greater feeding difficulties in premature infants born with very low birth weight, when compared to those born at term, which can persist in the long term, during and after the introduction of complementary feeding^(7)^. Invasive orofacial procedures, such as orotracheal intubation and gastric probing, to which premature infants are often subjected, provide negative stimulation to oral sensory and motor functions, and can generate adverse reactions when food is introduced at a later stage^(8,9)^. Therefore, it has been observed that the process of introducing food is not always well received by premature newborns, and difficulties in the transition to new food consistencies are demonstrated by refusing, vomiting, crying, irritability, nausea, and choking, which are frequent in this population^(10)^.

Despite the literature reporting such difficulties in preterm infants, there is still a lack of longitudinal studies covering the progression of food consistencies during the complementary feeding period, as well as the age at which they begin in the first year of life^(11)^. Most studies describe indicative signs of difficulties, such as early weaning and the introduction of early complementary feeding, but few studies assess and relate the oral function of these patients longitudinally^(12-16)^.

### Objective

Thus, this review aims to systematically review the literature regarding the impact of prematurity on the transition of food consistencies in infants during the introduction of complementary feeding.

## RESEARCH STRATEGY

This study is a systematic review conducted and reported according to the Preferred Reporting Items for Systematic Reviews and Meta-Analysis Checklist (PRISMA) 2020^(17)^. This study protocol was submitted and registered in the International Prospective Register of Systematic Reviews (PROSPERO) under the number CRD42020192884^(17)^.

## INCLUSION CRITERIA

The ‘PECOS’ criteria were applied to answer the following question: “Does prematurity impact the transition of food consistencies in infants during the introduction of complementary feeding?”

Population (P): Infants; Exposure (E): Prematurity; Comparison (C): Full-term newborns; Outcome (O): progression of food consistencies in premature infants with or without comparison; Study design (S): Cohort study, Case-control, Cross-sectional.

Studies with newborns with a gestational age (GA) of less than 37 weeks, that is, preterm infants without comorbidities and/or orofacial alterations that could interfere with the feeding process were included. The studies with or without comparisons with full-term newborns in the same study were also included. The studies needed to address the progression of food consistencies during the introduction of complementary feeding and present an analytical (observational) design. There was no restriction on ethnicity or gender, as well as the year of publication or language.

Studies with the following characteristic were excluded:

1- Studies with premature infants with craniofacial anomalies, genetic syndromes, neuromuscular diseases, cerebral palsy, and/or dysphagia
2- Studies with children over 24 months
3- Studies with no premature infants
4- Studies with infants with gestational age over 37 weeks, except those compared.
5- Studies without focus on the progression of food consistencies in preterm infants, with or without comparison.
6- Descriptive studies, such as letters to the editor, commentaries, case reports, expert opinions, conference abstracts, letters, posters, reviews, and books.
7- Studies conducted during the newborn hospitalization period without follow-up.
8- Articles with incomplete data

Appropriate word combinations and truncations were selected and tailored specifically for each electronic database: EMBASE, Latin American and Caribbean Literature in Health Sciences (LILACS), LIVIVO, PubMed/Medline, Scopus, and Web of Science (Appendix A).

Gray literature searches were also conducted on Google Scholar, Open Grey, and ProQuest Dissertations & Theses. After searching the electronic databases, a manual search of the references of the included studies was performed to include further relevant studies. The EndNote® reference manager (Thomson Reuters, Philadelphia, PA) was used to remove duplicate studies. The surveys were conducted on August 10, 2020, and updated on September 19, 2022.

The selection of studies was performed in two phases. In Phase 1, the titles and abstracts of all electronic databases were read. All articles that did not meet the eligibility criteria were excluded at this stage. In Phase 2, all selected studies were read in full, and the eligibility criteria was reapplied by the same reviewers. In both phases, the readings were blinded and independently performed by two reviewers (H.N.C.L.G and R.H.P.). Any disagreement or conflict between the two reviewers in phases 1 and 2 were discussed until a mutual agreement was reached; in cases of no consensus, a third reviewer (S.T.A.) was consulted for a final decision.

Before starting the Phase 1 reading, both reviewers were calibrated using the Kappa concordance index. Reading was only started after obtaining an index > 0.7, indicating good inter-reviewer agreement. The Rayyan website (https://rayyan.qcri.org/) was used to read, thus ensuring adequate blinding of the reviewers and greater transparency during these stages.

## DATA ANALYSIS

Two reviewers (H.N.C.L.G and R.H.P) independently selected and extracted data from the included articles and compared the extracted information. Any disagreement about the data was discussed among them and, if necessary, a third reviewer (S.T.A.) was consulted. The following data were extracted from the included articles: author; year of publication; country; study objective, sample characteristics (sample size, age, gender, progression of food consistencies), study design, results, and conclusion. In cases of missing or incomplete data in the article, three attempts were made to contact the authors by e-mail to obtain such information, with an interval of one week.

The methodological quality of the selected observational studies was assessed using the Meta-Analysis of Statistics Assessment and Review Instrument (MASTARI) Two reviewers (H.N.C.L.G and R.H.P) independently evaluated the risk of bias and categorized each article based on their assessment criteria: “high” if the study received a “yes” score below 49%, “moderate” if the score ranged from 50% to 69%, and “low” if it exceeded 70% of “yes” scores for risk of bias questions. When necessary, disagreements were discussed with a third reviewer (S.T.A.).

## RESULTS

**Figure 1:**
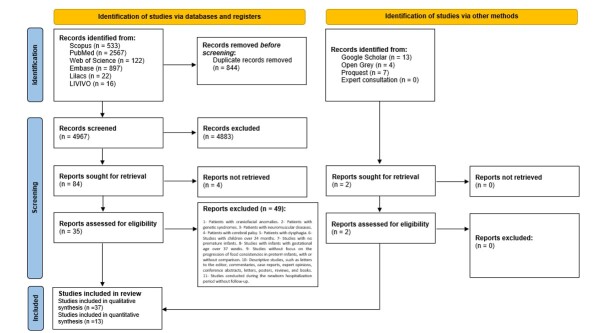
PRISM 2020 flow diagram for new systematic reviews that included searches on databases, registers, and other sources^(17)^

The database search resulted in 3,310 studies. Titles and abstracts were read (Phase 1), and 3,195 studies were excluded after resolving conflicts and doubts, as well as excluding ten duplicate studies. A total of 46 articles were selected to be read in full. The gray literature search was conducted on Google Scholar, Open Gray, and Proquest (Theses and Dissertations), identifying 78 studies; however, only one was selected. A manual search was performed in the references of the 46 studies selected for Phase 2 and three additional studies were identified. Thus, 50 studies in Phase 2 were selected for the full-text readings, and 41 were removed (Appendix B), totaling nine studies for the qualitative synthesis (Figure 1).

Regarding the study design, all nine articles were observational, with three cross-sectional studies^(10,11,18)^, five cohorts^(15,19-22)^, and one case-control^(8)^, published from 2004^(8)^ to 2020^(22).^

Of the nine studies, five of them were in English^(8,15,18,19,22)^ and four in Portuguese^(10,11,20,21)^; moreover, one was from England^(18)^, four from Brazil^(10,11,20,21)^, two from Australia^(8,15)^, one from the United States^(19)^, and one from Germany^(22)^.

The sample size ranged from 15^(18)^ to 85 preterm newborns (15), aged from zero to 24 months of corrected age.

Regarding the age at which complementary feeding was introduced, seven of the nine studies^(10,11,15,19-22)^ reported that it occurred early, i.e., before the recommended age of six months of corrected age and the appearance of signs of readiness^(23).^

Another described finding was the presence of oral motor dysfunction in the skills with cups, small pieces, and solids, showing gagging and defensive behaviors, i.e., difficulties related to grasp an open cup with the lips and to chew small pieces and solids^(8,10,11,18-22)^.

A study found a lower trend of food refusal in exclusively breastfed infants up to 6 months of age^(11)^. The same study identified an association between GA < 28 weeks, time of enteral and parenteral nutrition, and feeding difficulties. In four^(19-22)^ studies, improvement in oral motor dysfunction was observed at 12 months, i.e., an improvement in the skills of chewing solid foods and drinking from an open cup. In one study^(19)^ involving a sample of 41 preterm infants, most newborns were bottle-fed. Two other studies^(20,21)^, with 45 and 52 preterm infants respectively, included the same population. Finally, another study^(22)^ followed 40 preterm infants, all of which had follow-up assessments up to 12 months of corrected age

As a method of data collection, all studies used a questionnaire for demographic and neonatal history data. For the assessment of oral motor skills, the most used protocol was the Schedule for Oral Motor Assessment (SOMA), employed in three out of the nine studies^(18,20,21)^. The other studies used different protocols, such as: Checklist of the Brazilian Society of Pediatrics^(11)^, Clinical Evaluation Protocol of Pediatric Dysphagia (PAD-PED)^(11)^, The Child Feeding Skills Checklist^(19)^, Neonatal Oral Motor Assessment Scale (NOMAS)^(22)^, Observation List for Spoon Feeding (OSF)^(22)^, Mastication Observation and Evaluation Instrument (MOE)^(22)^, Royal Children’s Hospital Oral Sensitivity Checklist (OSC)^(8)^, and Pre-Speech Assessment Scale (PSAS)^(8)^ were used in one study^(15)^. However, in another study^(15)^, no specific protocol for oral motor assessment was used; only a structured questionnaire developed by the authors was employed.

Among the three cross-sectional observational studies included, one^(18)^ showed a low risk of bias and two^(10,11)^ presented a moderate risk. All three of these^(10,11,18)^ were negatively evaluated since they did not present a random sample. In two^(10,11)^ studies, the interfering factors were not recognized and the approaches to dealing with them were mentioned or were not explicit; in addition, the results of the participants who withdrew were not detailed and considered in the evaluation. In five observational cohort studies, there was a^(15)^ moderate risk, as the results were not assessed using objective criteria, the results of people who withdrew were not described and included in the analysis, and/or the results were not measured reliably. The other four^(19-22)^ presented low risk of bias. The only case-control study^(8)^ included presented low risk of bias (Appendix C).

Chart 1 shows the data extracted from each study included. In the selected studies, there was a tendency for solid foods to be introduced later, considering chronological age, among preterm infants when compared to term infants; however, when corrected age was considered, solid foods were introduced earlier in preterm infants^(10,11,15)^.

**Chart 1:** Characteristics of the included studies (n = 9)

Author (Country)	Sample size	Age, sex	Study design	Objective	Results and Conclusion
Brusco and Delgado^(10)^ (Brazil)	32 preterm infants	Mean: 4 months 27 days and CA: 1 month 27 days	Cross-sectional	To characterize the feeding development of preterm infants from three to 12 months, checking the breastfeeding type, the timing of introduction of complementary feeding, the deleterious oral habits, the guidance received, the feeding difficulties, and the sociodemographic profile.	Fluid supply observed early.
		Min: 90 days and Max: 10 months 29 days			Pasty food started at an appropriate age, but solids at an early age, 17 reported feeding difficulties. There was an association between refusal and GA and underweight, as well as hypotonia of the lip, tongue, and cheek with GA.
		21 males			
		11 females			
Buswell et al.^(18)^ (England)	15 preterm infants	10 months CA	Cross-sectional	To determine the presence of OMD during feeding in early childhood and whether neonatal factors associated with feeding difficulties are predictors of OMD	3 children had borderline or indicative OMD scores, 1 child had BMD for all consistencies, 2 had OMD for all except solids, and 3 did not eat solids.
		9 males			12 infants accepted all consistencies and there was no relationship between neonatal variables and the SOMA score.
		6 females			
Cleary et al.^(15)^ (Australia)	85 preterm infants	21 weeks preterm infant	Prospective cohort	To determine the age of introduction of solid foods in preterm infants, compared to preterm infants and associated factors.	They found early introduction of solids in preterm infants.
	65 full-term infants	19 weeks full-term infants			Type of breastfeeding and birth weight were not associated with the age at which solids were introduced.
		56 males			In full-term infants, lower maternal education and lower maternal age were associated with early introduction of solids.
		29 females			OMD was not evaluated in this study.
Dodrill et al.^(8)^ (Australia)	20 preterm infants	Mean 11-17 months CA	Case-control	To verify differences in oral sensitivity and feeding development between preterm and full-term infants and to examine differences in oral sensitivity and feeding development between infants who received shorter and longer periods of nasogastric feeding.	Differences were observed between preterm infants and full-term infants in behaviors suggestive of altered oral sensitivity (p = 0.000), and preterm infants had more tongue protrusion (p = 0.010) and escape when swallowing solids (p = 0.006).
	10 full-term infants	Females: 6 full-term infants and 11 preterm infants			
		Males: 4 full-term infants and 9 preterm infants			
Ferreira^(20)^ (Brazil)	45 preterm infants	M1 32.9 weeks	Prospective cohort	To verify the nutritional status and development of oral motor skills in preterm infants during the first year of life and the possible associations between them.	At 4 months, oral motor dysfunction was observed in 78% for the puree consistency, and at 6 months it decreased to 41%.
		M2 37.2 weeks			In the semi-solid consistency at six months, OMD was observed in 9% and none for liquids.
		M3 4.1 months			At 9 months, the OMD for liquids in cups was 14%. At 12 months, the OMD for liquids and solids was 13% and 9%, respectively.
		M4 6 months			
		M5 8.9 months			
		M6 11.9 months			
		24 females / 21 males			
Hübl et al.^(22)^ (Germany)	40 preterm infants	6/9/12/24 months	Prospective cohort	Examine early OMD and relate to difficulties in feeding semi-solids and solids	Food introduction was started with puree by all and at a lower age than recommended.
		21 females			Half of the infants received semi-solid and solid foods before the recommended age.
		19 males			At 12 months (postmenstrual age), 6 infants were not receiving solids due to choking and parental fear. In the evaluation of spoon feeding at 6 months, 10% achieved a total score; 9 months, 65.8%; and 12 months, 71.8%. Improvement in chewing was observed from 9 to 12 months and from 9 to 24 months.
Pridham et al.^(19)^ (United States)	41 preterm infants	Mean Min 40.2 Max 375.1 days	Prospective cohort	To examine the independent and interactive contribution of biological and maternal infant feeding conditions to feeding skill performance	Only 51% showed a sign of readiness for food introduction.
		1 av. 22 males and 22 females			At 8 months, 60% showed cup skills and coordination for grainy foods.
		2 av. 19 males and 16 females			At 12 months, 70% had the ability to eat all consistencies.
		3 av. 21 males and 17 females			
		4 av. 21 males and 20 females			
Steinberg et al.^(11)^ (Brazil)	62 preterm infants	Mean 13.5 months CA	Cross-sectional	To investigate whether there is an association between oral motor dysfunction and feeding difficulties during the process of introducing complementary feeding to preterm infants.	27 mothers reported difficulty in feeding their children. After applying the checklist, this number increased, and 43 of them reported that their child had some difficulty.
		36 females			Children who were exclusively breastfed up to 6 months had less tendency to refuse feeding.
		26 males			The most frequent defensive behavior was refusal to open the mouth. No association was observed between oral motor difficulty and feeding difficulty.
					However, there was an association between liquefied food, GI, nasogastric feeding tube time, and feeding difficulties.
Yamamoto^(21)^ (Brazil)	52 preterm infants	4/6/9/12 months	Prospective cohort	To investigate whether there is an association between oral motor dysfunction and feeding difficulties during the process of introducing complementary feeding to preterm infants.	At 4 months, most of them had oral motor dysfunction, and no association was observed between the result of the SOMA for puree and gestational age. At 6 months, 65.6% had normal oral motor function for puree, and 97% for semisolids. At 9 and 12 months, more than 85% had normal oral motor function for all consistencies.
		28 females			
		24 males			

Caption: CA = Corrected Age; GA = Gestational Age; OMD = Oral Motor Dysfunction

Source: The authors (2021)

There was also a relationship between invasive oral interventions and feeding difficulties, with p < 0.05 in all skills evaluated^(11)^.

The association between gestational age and feeding difficulties was statistically significant, showing that lower gestational age increased the frequency of feeding difficulties^(8,10,11)^. Gestational age-related oral motor dysfunction has been described in two studies^(10,21)^. An association was also found between oral motor dysfunction, gestational age, and solid consistency^(21)^, with the same relationship being observed in another study^(18)^.

Regarding the progression of consistencies, when comparing assessments at four and six months of corrected age, frequency of oral motor dysfunction for the pureed consistency decreases significantly^(20)^. Improvements in skills were also seen when evaluations were compared at nine and 12 months, and at nine and 24 months^(19,22)^.

None of the studies mention or infer that the participants underwent any kind of intervention, either guidance or rehabilitation.

Due to the methodological heterogeneity between studies and the diversity of assessment protocols used, it was not possible to conduct a quantitative synthesis. The only protocol used in at least three studies was the Schedule for Oral Motor Assessment (SOMA) protocol. However, of these three studies, two shared the same sample, so quantitative synthesis was not recommended to avoid duplicating data.

It is estimated that more than 15 million premature infants are born every year worldwide and, due to the technological quality of care provided to these newborns, an increase in survival has been noted. This increase, however, has led to further costs due to developmental delays, feeding difficulties, and other comorbidities affecting this population^(24)^.

The Brazilian Ministry of Health and the Brazilian Society of Pediatrics recommend that complementary feeding should be initiated at 6 months of age for full-term newborns^(25)^ and for preterm newborns, the introduction is recommended at six months corrected age^(23)^.

In addition to age, neurodevelopment in the baby must be considered, as well as corrected age for premature babies^(26)^. However, this practice was not observed in the studies in this review, which revealed that complementary feeding was introduced early in seven of the nine studies, at around four months of corrected age^(10,11,15,19-22)^. It was also observed that preterm infants were introduced to complementary feeding later than full-term infants, with a significant difference (p < 0.02)^(15)^, when not considering corrected age. When age is corrected, solid foods are introduced earlier in premature infants, with a significant difference (p < 0.001). This is relevant, as studies comparing the introduction of food at four months and six months observe an increased risk of infections when complementary feeding is introduced at four months^(24)^, as well as an increased risk of food allergies and obesity^(27)^.

It is also worth mentioning that there is no consensus in the literature as to when the introduction of complementary feeding should begin in premature infants. The European Society for Paediatric Gastroenterology Hepatology and Nutrition (ESPGHAN), as well as the World Health Organization, strongly recommend feeding breast milk to premature babies, emphasizing the importance of supporting and monitoring the mother/infant bond to qualify and expand breastfeeding time. Moreover, considering that this population is at increased nutritional risk, an individualized approach according to the infant’s neurological capacity and nutritional status would be indicated for a more assertive practice in the introduction of complementary feeding in premature infants, especially in the absence of evidence-based guidelines^(24)^.

In this review, one study^(11)^ showed a relationship between invasive oral interventions and feeding difficulties, with statistical significance in all the skills assessed, corroborating other studies that report a risk of feeding difficulties resulting from interventions that premature infants were subjected to while still in the hospital intensive care unit^(28)^. These interventions promote oral stimuli that cause a deficit in adequate sensory experience in the first few months, resulting in exacerbated and/or suppressed oral reflexes^(29)^. It is worth emphasizing that complementary feeding in the first years of life involves numerous factors that influence both the beginning and progression of food consistencies, and that monitoring with validated protocols is recommended, anticipating the various interfering biases to better understand these relationships.

Fine and gross motor skills, which are acquired with adequate neuropsychomotor development, constitute part of eating skills^(30)^. Delay in the acquisition of these skills is frequently observed in preterm infants. Only one study found no association between gestational age and oral motor dysfunction (OMD)^(18)^, and this was a study with a small sample of 36 infants with gestational age < 37 weeks at birth. Meanwhile, three other studies show that lower gestational age leads to more frequent eating difficulties, with statistical significance^(8,10,11)^.

OMD was related to gestational age in two studies^(10,21)^. In both cases, the study population was composed of premature infants who had undergone speech-language pathology evaluation. However, the first study monitored extreme and late preterm infants from birth to 12 months, while the second conducted a cross-sectional study of late preterm infants. However, in the latter, sampling was not performed randomly.

Another study^(12)^ which administered a questionnaire to parents of children at the age of two found that 14.9% of late and moderate preterm infants had feeding difficulties compared to 9.5% of full-term infants, which represents a 57% increase in feeding difficulties among preterm babies, including oral motor problems.

Regarding the progression of food consistencies, an improvement in oral skills was observed with the pureed consistency from four to six months of corrected age^(20)^. Moreover, an improvement in skills was found when comparing nine months and 12 months, and nine months and 24 months, with p = 0.001^(19,22)^. These data agree with the findings of a review in which mature eating skills were observed to occur alongside anatomical changes during growth and neuropsychomotor development, as well as experiences with various types of food, textures, and their presentation in the first years of life^(7)^. This period provides infants with new experiences with food textures and flavors, contributing to the modulation and brain connections responsible for controlling intake, with long-term outcomes^(13)^.

However, the studies did not mention whether speech-language therapy intervention was conducted, which may influence the frequency of difficulties, given that specialized monitoring, guidance, and even interventions to address initial difficulties in the introduction of complementary feeding may prevent this population from experiencing delays.

It is also important to consider that the study protocols varied, as well as their instruments and methods to assess problems in the progression of food consistencies during the introduction of complementary feeding. This highlights that an ideal instrument for assessing feeding skills in this age group does not yet exist, and that there is a need for a consensus to standardize more assertive assessments.

## CONCLUSION

Most studies found no association between prematurity and difficulties with the progression of food consistencies during the introduction of complementary feeding. Overall, only three studies showed an association with feeding difficulties, characterized by refusal to feed, refusal to open the mouth, vomiting, and defensive signs during feeding.

Some studies pointed to an improvement in oral skills as preterm infants grow and show signs of readiness, with consequent improvement in skills for semi-solid and solid consistencies from 12 to 24 months.

The selected studies showed methodological heterogeneity, including varying protocols.

We emphasize the importance of standardizing screening instruments and conducting further studies on the assessment of oral motor dysfunction in preterm infants and comparing them with full-term infants to verify the need for early monitoring and intervention to prevent feeding, nutritional, and neuropsychomotor developmental difficulties in preterm infants.

## Appendix A Database search strategy

**Table:**

Database	Search (April 24^th^, 2020)
LILACS	(“premature birth” OR “Very preterm infant OR “Premature Births” OR “Preterm Birth” OR “Preterm Births” OR “Premature Infant” OR “Very preterm birth” OR “Preterm Infants” OR “Preterm Infant” OR “Premature Infants” OR “nascimento premature” OR “Bebê muito premature” OR “Nascimentos prematuros” OR “Bebê premature” OR “Nascimento muito premature” OR “Bebês prematuros” OR “nacimiento premature” OR “bebé muy premature” OR “nacimientos prematuros” OR “bebé premature” OR “nacimiento muy premature” OR “bebés prematuros”) AND (“Feeding Behavior” OR “Feeding Behaviors” OR “Eating Behavior” OR “Eating Behaviors” OR “oral feeding progression” OR “solid food introduction” OR “past food introduction” OR “Comportamento alimentar” OR “Comportamentos alimentares” OR “progressão da alimentação oral” OR “introdução de alimento sólido” OR “Comportamiento alimenticio” OR “Comportamientos alimentícios” OR “progresión de la alimentación oral” OR “introducción de alimentos sólidos”)
PubMed	1. (“premature birth”[MeSH Terms] OR “premature birth”[All Fields] OR “Very preterm infant”[All Fields] OR “Premature Births”[All Fields] OR “Preterm Birth”[All Fields] OR “Preterm Births”[All Fields] OR “Premature Infant”[All Fields] OR “Very preterm birth”[All Fields] OR “Preterm Infants”[All Fields] OR “Preterm Infant”[All Fields] OR “Premature Infants”[All Fields]) 2. (“feeding behavior”[MeSH Terms] OR “feeding behavior”[All Fields] OR “Feeding Behaviors”[All Fields] OR “Eating Behavior”[All Fields] OR “Eating Behaviors”[All Fields] OR “oral feeding progression”[All Fields] OR “solid food introduction”[All Fields]) 3. #1 AND #2
SCOPUS	TITLE-ABS-KEY(“premature birth” OR “Very preterm infant” OR “Premature Births” OR “Preterm Birth” OR “Preterm Births” OR “Premature Infant” OR “Very preterm birth” OR “Preterm Infants” OR “Preterm Infant” OR “Premature Infants”) AND TITLE-ABS-KEY(“Feeding Behavior” OR “Feeding Behaviors” OR “Eating Behavior” OR “Eating Behaviors” OR “oral feeding progression” OR “solid food introduction” OR “past food introduction”)
Web of Science	8. TS=(“premature birth” OR “Very preterm infant” OR “Premature Births” OR “Preterm Birth” OR “Preterm Births” OR “Premature Infant” OR “Very preterm birth” OR “Preterm Infants” OR “Preterm Infant” OR “Premature Infants”) 9. TS=(“Feeding Behavior” OR “Feeding Behaviors” OR “Eating Behavior” OR “Eating Behaviors” OR “oral feeding progression” OR “solid food introduction” OR “past food introduction”) 10. #1 AND #2
LIVIVO	TI=(“premature birth” OR “Very preterm infant” OR “Premature Births” OR “Preterm Birth” OR “Preterm Births” OR “Premature Infant” OR “Very preterm birth” OR “Preterm Infants” OR “Preterm Infant” OR “Premature Infants”) AND TI=(“Feeding Behavior” OR “Feeding Behaviors” OR “Eating Behavior” OR “Eating Behaviors” OR “oral feeding progression” OR “solid food introduction” OR “past food introduction”)
EMBASE	(‘premature birth’/exp OR ‘premature birth’ OR ‘very preterm infant’/exp OR ‘very preterm infant’ OR ‘premature births’ OR ‘preterm birth’/exp OR ‘preterm birth’ OR ‘preterm births’ OR ‘premature infant’/exp OR ‘premature infant’ OR ‘very preterm birth’/exp OR ‘very preterm birth’ OR ‘preterm infants’ OR ‘preterm infant’/exp OR ‘preterm infant’ OR ‘premature infants’) AND (‘feeding behavior’/exp OR ‘feeding behavior’ OR ‘feeding behaviors’ OR ‘eating behavior’/exp OR ‘eating behavior’ OR ‘eating behaviors’ OR ‘oral feeding progression’ OR ‘solid food introduction’ OR ‘past food introduction’)
Google Scholar	“premature birth” AND “feeding behavior”
Open Grey	“premature birth”
ProQuest	(“premature birth” OR “Very preterm infant” OR “Premature Births” OR “Preterm Birth” OR “Preterm Births” OR “Premature Infant” OR “Very preterm birth” OR “Preterm Infants” OR “Preterm Infant” OR “Premature Infants”) AND (“Feeding Behavior” OR “Feeding Behaviors” OR “Eating Behavior” OR “Eating Behaviors” OR “oral feeding progression” OR “solid food introduction” OR “past food introduction”)

Source: The authors (2021)

## Appendix B Excluded articles and the reasons for exclusion (n= 41)

**Table:**

Author, year	Reasons for exclusion
A. Kirk, S. Alder, J. King; 2006	11
I. Adams-Chapman, C.M. Bann, Y.E. Vaucher, MD, B.J. Stoll; 2013	5
R. Barachetti, E. Villa, M. Barbarini; 2017	10
E. N. Bezze, M.L. Giannì, P. Sannino, C. Esposito, L. Plevani, S. Muscolo, P. Roggero, F. Mosca; 2017	10
J.V. Browne, E.S. Ross; 2011	10
A.N. Coşkun, P.Z. Akkuş, E.I. Bahadur, H.T.Çelik, E.N. Özmert, 2019	10
T.L. Crapnell, C.E. Rogers, J.J. Neil, T.E. Inder, L.J. Woodward, R.G. Pineda, 2013	9
Crapnell, T.L., Woodward, L.J., Rogers, C.E., Inder, T.E., Pineda, R.G., 2015	9
Delaney, A.L., Arvedson, J.C., 2008	10
DeMauro SB, Patel PR, Medoff-Cooper B, Posencheg M, Abbasi S., 2011	9
S.L. den Boer, J.A. Schipper, 2013	9
P. Dodrill,; T. Donovan; 2014	10
N.D. Embleton, M. Fewtrell, 2017	10
S. Fanaro, G. Borsari, V. Vigi, 2007	9
Giannì M.L., Bezze E, Colombo L, Rossetti C, Pesenti N, Roggero P, Sannino P, Muscolo S, Plevani L, Mosca F., 2018	9
Howe T.H., Sheu C.F., Wang T.N., 2019	9
S. Johnson, R. Matthews, E.S. Draper, D.J. Field, B.N. Manktelow, N.Marlow, L.K. Smith, E.M. Boyle, 2016	9
Kennedy C., Lipsitt L.P., 1993	10
King, C., 2009	10
Méio M.D.B.B., Villela L.D., Gomes Júnior S.C.D.S., Tovar C.M., Moreira M.E.L., 2018	9
Menezes LVP, Steinberg C, Nóbrega AC., 2017	9
Migraine A, Nicklaus S, Parnet P, Lange C, Monnery-Patris S, Des Robert C, Darmaun D, Flamant C, Amarger V, Rozé JC., 2013	9
Navarro, L.; Antunes, H.; 2019	7
Norris, F., Larkin, M., Williams, C. Hampton, S.M. Morgan, J.B., 2002	9
O'Grady, R. S.; 1971	10
Palmer D.J., Makrides M., 2012	10
Patra, K., Greene, M.M., 2019	9
Philip, A. K.; Vijay Kumar, K. V., 2015	9
Pineda, R. G., 2016	10
Pridham, K.; Saxe, R.; Limbo, R.; 2004	10
Rodriguez J, Affuso O, Azuero A, Downs CA, Turner-Henson A, Rice M., 2018	9
Ross, E. S.; Browne, J. V.; 2002	10
Silberstein, D.; Feldman, R.; Gardner, J. M.; Karmel, B. Z.; Kuint, J.; Geva, R.; 2009	9
Törölä H, Lehtihalmes M, Yliherva A, Olsén P. 2012	10
van Dijk M, Bruinsma E, Hauser MP, 2016	9
Yrjänä JMS, Koski T, Törölä H, Valkama M, Kulmala P. 2018	9
Zielinska MA, Rust P, Masztalerz-Kozubek D, Bichler J, Hamułka J. 2019	9
Cerro N.,Zeunert S., Simmer KN, Daniels A., 2002	9
Pridham K., Brown R., Clark R., Limbo RK., 2005	9
Fewtrell M., Lucas A., Morgan JB., 2018	10
Chung, J.; Lee, J.; Spinazzola, R.; Rosen, L.; Milanaik, R.; 2014	9

Caption: 1- Patients with craniofacial anomalies. 2- Patients with genetic syndromes; 3- Patients with neuromuscular diseases; 4- Patients with cerebral palsy; 5- Patients with dysphagia; 6- Studies with children over 24 months; 7- Studies with no premature infants; 8- Studies with infants with gestational age over 37 weeks; 9- Studies without focus on the progression of food consistencies in preterm infants, with or without comparison; 10- Descriptive studies, such as letters to the editor, commentaries, case reports, expert opinions, conference abstracts, letters, posters, reviews, and books; 11- Studies conducted during the newborn hospitalization period without follow-up

References

1 - Kirk A, Alder S, King J. 109 Risk factors for poor oral feeding progression in premature infants. Journal of Investigative Medicine 2006;54:S98.

2 - Adams-Chapman I, Bann CM, Vaucher YE, Stoll BJ; Eunice Kennedy Shriver National Institute of Child Health and Human Development Neonatal Research Network. Association between feeding difficulties and language delay in preterm infants using Bayley Scales of Infant Development-Third Edition. J Pediatr. 2013;163(3):680-5.e53.

3 - Barachetti R, Villa E, Barbarini M. Weaning and complementary feeding in preterm infants: management, timing and health outcome. Pediatr Med Chir. 2017 Dec 22;39(4):181. doi: 10.4081/pmc.2017.181. PMID: 29502384.

4 - Bezze EN Giannì ML, Sannino P, Esposito C, Plevani L, Muscolo S. *et al*. The assessment of oral feeding skills in preterm infants. Pediatria Medica e Chirurgica. 2017; 39(1): 17.

5 - Browne JV, Ross ES. Eating as a Neurodevelopmental Process for High-Risk Newborns. Clinics in Perinatology. 2011; 38 (4): 731-743.

6 – Coşkun AN, Akkuş PZ., Bahadur EI, Çelik HT, Özmert EN, Early feeding problems and associated factors in premature infants. Archives of Disease in Childhood. 2019; 104 (0): A208-A209.

7 – Crapnell, TL., Rogers, CE., Neil, JJ., Inder, TE., Woodward, L.J., Pineda, R.G. Factors associated with feeding difficulties in the very preterm infant. Acta Paediatrica, 2013 Dec;102(12):e539-45.

8 - Crapnell TL, Woodward LJ, Rogers CE, Inder TE, Pineda RG. Neurodevelopmental Profile, Growth, and Psychosocial Environment of Preterm Infants with Difficult Feeding Behavior at Age 2 Years. J Pediatr. 2015 Dec;167(6):1347-53.

9 - Delaney AL, Arvedson JC. Development of swallowing and feeding: prenatal through first year of life. Dev Disabil Res Rev. 2008;14(2):105-17. doi: 10.1002/ddrr.16. PMID: 18646020.

10 - DeMauro SB, Patel PR, Medoff-Cooper B, Posencheg M, Abbasi S. Postdischarge feeding patterns in early- and late-preterm infants. Clin Pediatr (Phila). 2011 Oct;50(10):957-62.

11 - den Boer SL, Schipper JA. Feeding and drinking skills in preterm and low birth weight infants compared to full term infants at a corrected age of nine months. Early Hum Dev. 2013 Jun;89(6):445-7. doi: 10.1016/j.earlhumdev.2012.12.004. Epub 2012 Dec 27.

12 - Dodrill P, Donovan T. Feeding skills and dietary intake of preterm infants at 12 months corrected age. Dysphagia. 2014; 29 (6): 758.

13 - Embleton ND, Fewtrell M. Complementary feeding in preterm infants. The Lancet. 2017; 5(5): 470-e471.

14 - Fanaro S, Borsari G, Vigi V. Complementary feeding practices in preterm infants: an observational study in a cohort of Italian infants. J Pediatr Gastroenterol Nutr. 2007 Dec;45 Suppl 3:S210-4.

15 - Giannì ML, Bezze E, Colombo L, Rossetti C, Pesenti N, Roggero P, Sannino P, Muscolo S, Plevani L, Mosca F. Complementary Feeding Practices in a Cohort of Italian Late Preterm Infants. Nutrients. 2018 Dec 2;10(12):1861.

16 - Howe TH, Sheu CF, Wang TN. Feeding Patterns and Parental Perceptions of Feeding Issues of Preterm Infants in the First 2 Years of Life. Am J Occup Ther. 2019 Mar/Apr;73(2):7302205030p1-7302205030p10.

17 – Johnson S, Matthews R, Draper ES, Field DJ, Manktelow BN, Marlow N. *et al*. Eating difficulties in children born late and moderately preterm at 2 y of age: a prospective population-based cohort study–, The American Journal of Clinical Nutrition. 2016; 103(2): 406–414

18 - Kennedy C, Lipsitt LP. Temporal characteristics of non-oral feedings and chronic feeding problems in premature infants. J Perinat Neonatal Nurs. 1993 Dec;7(3):77-89.

19 - King, C. An evidence based guide to weaning preterm infants. Symposium: Nutrition. Paediatrics and Child Health.2009; 19(9): 405-414.

20 - Méio MDBB, Villela LD, Gomes Júnior SCDS, Tovar CM, Moreira MEL. Breastfeeding of preterm newborn infants following hospital discharge: follow-up during the first year of life. Cien Saude Colet. 2018; 23 (7): 2403-2412.

21 – Menezes LVP, Steinberg C, Nóbrega AC. Complementary feeding in infants born prematurely. Codas. 2018; 30(6): e20170157.

22 - Migraine A, Nicklaus S, Parnet P, Lange C, Monnery-Patris S, Des Robert C, Darmaun D, Flamant C, Amarger V, Rozé JC. Effect of preterm birth and birth weight on eating behavior at 2 y of age. Am J Clin Nutr. 2013 Jun;97(6):1270-7.

23 - Navarro L, Antunes H, Baby-led weaning: A trend in complementary feeding? A population based cross-sectional study. Journal of Pediatric Gastroenterology and Nutrition. 2019; 68 (0): 352, 2019.

24 - Norris F, Larkin M, Williams C, Hampton SM, Morgan JB. Factors affecting the introduction of complementary foods in the preterm infant. Eur J Clin Nutr. 2002; 56: 448–454.

25 - O'Grady RS. Feeding behavior i infants. Am J Nurs. 1971; 71 (4): 736-9.

26 - Palmer DJ, Makrides M. Introducing solid foods to preterm infants in developed countries. Ann Nutr Metab. 2012; 60 (2):31-8.

27 - Patra K, Greene MM. Impact of feeding difficulties in the NICU on neurodevelopmental outcomes at 8 and 20 months corrected age in extremely low gestational age infants. J Perinatol. 2019 Sep;39(9):1241-1248.

28 - Philip AK, Vijay Kumar KV. Comparison of feeding behaviours in term infants and preterm infants (30 to 34 weeks) at six months corrected age. Journal of Nepal Paediatric Society. 2015; 35 (2): 202-205.

29 - Pineda RG. Feeding: an important, complex skill that impacts nutritional, social, motor and sensory experiences. Acta Paediatrica, International Journal of Paediatrics. 2016; 105 (10): e458.

30 - Pridham K, Saxe R, Limbo R, Feeding issues for mothers of very low-birth-weight, premature infants through the first year. J Perinat Neonatal Nurs. 2004; 18 (2): 161-9.

31 - Rodriguez J, Affuso O, Azuero A, Downs CA, Turner-Henson A, Rice M. Infant Feeding Practices and Weight Gain in Toddlers Born Very Preterm: A Pilot Study. J Pediatr Nurs. 2018; 43: 29-35.

32 - Ross ES, Browne JV. Developmental progression of feeding skills: an approach to supporting feeding in preterm infants. Semin Neonatol. 2002; 7 (6): 469-75.

33 - Silberstein D, Feldman R, Gardner JM, Karmel BZ, Kuint J, Geva R. The mother-infant feeding relationship across the first year and the development of feeding difficulties in low-risk premature infants. Infancy. 2009; 14 (5): 501-525.

34 - Törölä H, Lehtihalmes M, Yliherva A, Olsén P. Feeding skill milestones of preterm infants born with extremely low birth weight (ELBW). Infant Behav Dev. 2012 Apr;35(2):187-94.

35 - van Dijk M, Bruinsma E, Hauser MP. The relation between child feeding problems as measured by parental report and mealtime behavior observation: A pilot study. Appetite. 2016 Apr 1;99:262-267.

36 - Yrjänä JMS, Koski T, Törölä H, Valkama M, Kulmala P. Very early introduction of semisolid foods in preterm infants does not increase food allergies or atopic dermatitis. Ann Allergy Asthma Immunol. 2018 Sep;121(3):353-359.

37 - Zielinska MA, Rust P, Masztalerz-Kozubek D, Bichler J, Hamułka J. Factors Influencing the Age of Complementary Feeding-A Cross-Sectional Study from Two European Countries. Int J Environ Res Public Health. 2019 Oct 9;16(20):3799.

38 – Cerro N, Zeunert S, Simmer KN, Daniels LA. Eating behaviour of children 1.5-3.5 years born preterm: parents' perceptions. J Paediatr Child Health. 2002 Feb;38(1):72-8. doi: 10.1046/j.1440-1754.2002.00728.x. PMID: 11869405.

39 - Pridham K, Brown R, Clark R, Limbo RK, Schroeder M, Henriques J, Bohne E. Effect of guided participation on feeding competencies of mothers and their premature infants. Res Nurs Health. 2005 Jun;28(3):252-67. doi: 10.1002/nur.20073. PMID: 15884024.

40 - Fewtrell MS, Lucas A, Morgan JB. Fatores associados ao desmame em bebês nascidos a termo e pré-termo. Arch Dis Child Fetal Neonatal Ed. 2003; 88 (4): F296-F301. doi: 10.1136 / fn.88.4.f296

41 - Chung J, Lee J, Spinazzola R, Rosen L, Milanaik R. Parental perception of premature infant growth and feeding behaviors: use of gestation-adjusted age and assessing for developmental readiness during solid food introduction. Clin Pediatr (Phila). 2014 Nov;53(13):1271-7. doi: 10.1177/0009922814540039. Epub 2014 Jun 24. PMID: 24961782.

## Appendix C Risk of BIAS for the studies included in the qualitative and quantitative analysis, via the Meta-Analysis of Statistics Assessment and Review Instrument (MASTARI). BIAS risk was categorized as follows: ‘High’ if the study received a ‘Yes’ score below 49%, ‘Moderate’ if the score ranged from 50% to 69%, and ‘Low’ if it exceeded 70% of ‘Yes’ scores for risk of bias questions

**3.1 -** Studies included in the qualitative analysis

**Table:** A – Cross-sectional.

Question					
	Buswell et al.^(18)^	Steinberg et al.^(11)^	Brusco and Delgado^(10)^		
1. Was the study based on a random or pseudorandom sample?	N	N	N		
2. Were the criteria for inclusion in the sample clearly defined?	Y	Y	Y		
3. Were confounding factors identified and strategies to deal with them stated?	Y	N	U		
4. Were outcomes assessed using objective criteria?	Y	Y	Y		
5. If comparisons are being made, was there sufficient description of the groups?	NA	NA	NA		
6. Was the follow up carried out over a sufficient time period?	Y	U	Y		
7. Were the outcomes of people who withdrew described and included in the analysis?	Y	N	N		
8. Were the outcomes measured in a reliable way?	Y	Y	U		
9. Was an appropriate statistical analysis used?	Y	Y	Y		
% yes/risk	87.5% Low	50% Moderate	50% Moderate		

Caption: Y = Yes; N = No; U = Unclear; NA = Not applicable

**Table:** B - Cohort study/Case-control study.

Question	Cleary et al.^(15)^	Pridham et al.^(19)^	Hübl et al.^(22)^	Ferreira^(20)^	Yamamoto^(21)^	Dodrill et al.^(8)^
1. Was the sample representative of patients in the population as a whole?	Y	Y	Y	Y	Y	N
2. Were the patients at a similar point in the course of their condition/illness?	Y	Y	Y	Y	Y	Y
3.Had bias been minimized in relation to selection of cases and of controls?	Y	Y	NA	Y	Y	Y
4. Were confounding factors identified and strategies to deal with them stated?	Y	Y	Y	Y	Y	Y
5. Were the outcomes assessed using objective criteria?	N	Y	Y	Y	Y	Y
6. Was follow-up carried out over a sufficient time period?	Y	Y	Y	Y	Y	Y
7. Were the outcomes of people who withdrew described and included in the analysis?	N	Y	Y	Y	Y	Y
8. Were outcomes measured in a reliable way?	N	Y	U	Y	Y	Y
9. Was appropriate statistical analysis used?	Y	Y	Y	Y	Y	Y
% yes/risk	6.6% Moderate	00%Low	00% Low	00% Low	00% Low	8.8% Low

Caption: Y = Yes; N = No; U = Unclear; NA = Not applicable

## References

[1] Brasil (2019). Informações de saúde.

[2] Michels KA, Ghassabian A, Mumford SL, Sundaram R, Bell EM, Bello SC (2017). Breastfeeding and motor development in term and preterm infants in a longitudinal US cohort. Am J Clin Nutr.

[3] Lau C (2016). Development of infant oral feeding skills: what do we know?. Am J Clin Nutr.

[4] Browne JV, Ross ES (2011). Eating as a neurodevelopmental process for high-risk newborns. Clin Perinatol.

[5] Morris SE, Klein MD (2000). Pre-feeding skills: a comprehensive resource for mealtime development..

[6] Araújo CMT (2004). Alimentação complementar e desenvolvimento sensório motor oral.

[7] Pagliaro CL, Bühler KE, Ibidi SM, Limongi SC (2016). Dietary transition difficulties in preterm infants: critical literature review. J Pediatr.

[8] Dodrill P, McMahon S, Ward E, Weir K, Donovan T, Riddle B (2004). Long-term oral sensitivity and feeding skills of low-risk pre-term infants. Early Hum Dev.

[9] Jonsson M, van doorn J, van den Berg J (2013). Parents’ perceptions of eating skills of pre-term vs full-term infants from birth to 3 years. Int J Speech Lang Pathol.

[10] Brusco TR, Delgado SE (2014). Caracterização do desenvolvimento da alimentação de crianças nascidas pré-termo entre três e 12 meses. Rev CEFAC.

[11] Steinberg C, Menezes L, Nóbrega AC (2021). Disfunção motora oral e dificuldade alimentar durante a alimentação complementar em crianças nascidas pré-termo. CoDAS.

[12] Johnson S, Matthews R, Draper ES, Field DJ, Manktelow BN, Marlow N (2016). Eating difficulties in children born late and moderately preterm at 2 y of age: a prospective population-based cohort study. Am J Clin Nutr.

[13] Giannì ML, Bezze E, Colombo L, Rossetti C, Pesenti N, Roggero P (2018). Complementary feeding practices in a cohort of italian late preterm infants. Nutrients.

[14] Menezes LVP, Steinberg C, Nóbrega AC (2018). Complementary feeding in infants born prematurely. CoDAS.

[15] Cleary J, Dalton SM, Harman A, Wright IM (2020). Current practice in the introduction of solid foods for preterm infants. Public Health Nutr.

[16] Hofstätter E, Köttstorfer V, Stroicz P, Schütz S, Auer-Hackenberg L, Brandner J (2021). Introduction and feeding practices of solid food in preterm infants born in Salzburg. BMC Pediatr.

[17] Page MJ, Moher D, Bossuyt PM, Boutron I, Hoffmann TC, Mulrow CD (2021). PRISMA 2020 explanation and elaboration: updated guidance and exemplars for reporting systematic reviews. BMJ.

[18] Buswell CA, Leslie P, Embleton ND, Drinnan MJ (2009). Oral-motor dysfunction at 10 months corrected gestational age in infants born less than 37 weeks preterm. Dysphagia.

[19] Pridham K, Steward D, Thoyre S, Brown R, Brown L (2007). Feeding skill performance in premature infants during the first year. Early Hum Dev.

[20] Ferreira PF (2016). Estado nutricional e desenvolvimento das habilidades motoras orais para a alimentação em crianças nascidas pré-termo.

[21] Yamamoto RCC (2017). Caracterização do desenvolvimento das habilidades motoras orais de crianças de 0 a 12 meses de idade nascidas pré-termo.

[22] Hübl N, Costa SPD, Kaufmann N, Oh J, Willmes K (2020). Sucking patterns are not predictive of further feeding development in healthy preterm infants. Infant Behav Dev.

[23] SBP: Sociedade Brasileira de Pediatria (2018). Manual de alimentação: orientações para alimentação do lactente ao adolescente, na escola, na gestante, na prevenção de doenças e segurança alimentar..

[24] Embleton ND, Fewtrell M (2017). Complementary feeding in preterm infants. Lancet Glob Health.

[25] Brasil (2019). Guia alimentar para crianças brasileiras menores de 2 anos..

[26] SBP: Sociedade Brasileira de Pediatria (2021). A criança prematura: suas peculiaridades e o papel da família..

[27] Fewtrell M, Bronsky J, Campoy C, Domellöf M, Embleton N, Fidler Mis N (2017). Complementary feeding: a position paper by the European Society for Paediatric Gastroenterology, Hepatology, and Nutrition (ESPGHAN) Committee on Nutrition. J Pediatr Gastroenterol Nutr.

[28] Ross ES, Browne JV (2013). Feeding outcomes in preterm infants after discharge from the neonatal intensive care unit (NICU): a systematic review. Newborn Infant Nurs Rev.

[29] Bage AV (1999). A conquista das habilidades de alimentação do recém-nascido prematuro.

[30] Thoyre SM, Shaker CS, Pridham KF (2005). The early feeding skills assessment for preterm infants. Neonatal Netw.

