# Supplementary material for: Relationship between prematurity and difficulties in the transition of food consistency in childhood: a systematic review
Source: Codas. 2024 May 31;36(4):e20230100. doi: 10.1590/2317-1782/20242023100en (PMC11296670; doi:10.1590/2317-1782/20242023100en)
Supplement: Translation

# Translation: Relação entre prematuridade e dificuldades na transição da consistência alimentar na infância: uma revisão sistemática

**DOI:** 10.1590/2317-1782/20242023100pt

**Authors:** Hellen Nataly Correia Lagos Guimarães, Renata Henrique Petreça, Sheila Tamanini de Almeida, Flavio Magno, Rosane Sampaio Santos, Karinna Veríssimo Meira Taveira, Cristiano Miranda de Araujo, Adriane Celli

**Affiliations:** 1 Programa de Pós-graduação em Saúde da Criança e do Adolescente, Universidade Federal do Paraná – UFPR - Curitiba (PR), Brasil.; 2 Departamento de Fonoaudiologia, Universidade Federal de Ciências da Saúde de Porto Alegre – UFCSPA - Porto Alegre (RS) Brasil.; 3 Programa de Distúrbios da Comunicação, Universidade Tuiuti do Paraná – UTP - Curitiba (PR), Brasil.; 4 Departamento de Morfologia, Universidade Federal do Rio Grande do Norte – UFRN - Natal, RN, Brasil.; 5 Programa de Pós-graduação em Saúde da Criança e do Adolescente, Universidade Federal do Paraná – UFPR - Curitiba (PR), Brasil.

**Keywords:** Alimentação Complementar, Comportamento Alimentar, Recém-Nascido Prematuro, Lactente, Fonoaudiologia

## Abstract

**Objetivo:**

Revisar a literatura de maneira sistematizada acerca da relação do impacto da prematuridade na transição das consistências alimentares em lactentes no período de introdução da alimentação complementar.

**Estratégia de pesquisa:**

Foram pesquisados os bancos de dados EMBASE, Literatura Latino-Americana e do Caribe em Ciências da Saúde (LILACS), LIVIVO, PubMed/Medline, Scopus e Web of Science e Google Scholar, Open Grey e ProQuest Dissertations & Theses na literatura cinzenta foram pesquisados desde 10/08/2020.

**Critérios de seleção:**

“PECOS”: População (P): Lactentes, Exposição (E): Prematuridade, Comparação (C): Recém-nascidos a termo, Desfecho (O): Progressão de consistências alimentares em recém-nascidos prematuros com ou sem comparação, Tipos de estudos (S): Estudo de coorte; Caso-controle; Transversal.

**Análise dos dados:**

A qualidade metodológica dos estudos observacionais selecionados foi avaliada usando a Meta-Analysis of Statistics Assessment and Review Instrument (MASTARI).

**Resultados:**

Um total de 3.310 artigos foram encontrados, sendo 9 selecionados para a realização da síntese qualitativa. Nos estudos selecionados foi observada a relação entre intervenções orais invasivas e dificuldade alimentar para todas as habilidades avaliadas e quanto menor a idade gestacional, mais frequente são os comportamentos de dificuldades alimentares.

**Conclusão:**

Não foi observada relação entre a prematuridade e dificuldades na progressão das consistências no período da introdução das consistências alimentares na maioria dos estudos, somente três deles demonstraram tal relação.

## INTRODUÇÃO

No Brasil nascem aproximadamente três milhões de recém-nascidos ao ano, mais especificamente 2.849.146, destes 11% são prematuros^(1)^. Com o avanço das tecnologias a sobrevida desta população vem aumentando, desta maneira, estes são privados do período de desenvolvimento cerebral intrauterino trazendo assim, consequências como o aumento na frequência de comorbidades e atrasos do desenvolvimento, além de dificuldades no que diz respeito à alimentação^(2,3)^.

A introdução da alimentação complementar em lactentes prematuros, aos seis meses de idade corrigida, melhora a aceitabilidade alimentar, pois os lactentes têm mais experiências sensoriais e estão mais organizados neurologicamente. Além disso os padrões de movimentos se desenvolvem do motor global para o motor fino, sendo assim entendendo que a habilidade do comer se trata de uma habilidade motora fina, o desenvolvimento motor global é essencial para uma boa função oral. Consequentemente a estabilidade oral depende do controle de cabeça e ombros, os quais se relacionam com a estabilidade de tronco e pelve, todas estas habilidades de controle do eixo estão relacionadas ao desenvolvimento motor amplo. Então, à medida que o desenvolvimento motor acontece, funções e movimentos complexos podem ser executados pela criança^(4-6)^.

Em uma revisão de literatura foram descritas dificuldades alimentares em lactentes prematuros nascidos com muito baixo peso, quando comparadas aos nascidos a termo, podendo persistir em longo prazo, durante e após a introdução alimentação complementar^(7)^. Os procedimentos orofaciais invasivos como por exemplo intubação orotraqueal e de sondagem gástrica aos quais estes são submetidos com frequência, proporcionam estimulação negativa em funções sensoriais e motoras orais e podem gerar reações adversas quando o alimento é introduzido em um estágio posterior^(8,9)^. Por conseguinte, observa-se que o processo da introdução alimentar nem sempre é bem tolerado nos recém-nascidos prematuros e dificuldades na transição alimentar para novas consistências são demonstradas por meio de recusa, vômito, choro, irritabilidade, náuseas e engasgos, frequentes nesta população^(10)^.

Embora a literatura relate tais dificuldades nos lactentes prematuros, ainda há uma escassez de estudos longitudinais que abordem a progressão das consistências alimentares no período de alimentação complementar, bem como a idade de início destas no primeiro ano de vida^(11)^. A maioria dos estudos descreve sinais indicativos de dificuldades, tais como o desmame precoce e a introdução da alimentação complementar precoce, entretanto poucos estudos avaliam e relacionam longitudinalmente a função oral destes pacientes^(12-16)^.

### Objetivo

Desta forma, a presente revisão tem como objetivo revisar a literatura de maneira sistematizada acerca da relação do impacto da prematuridade na transição das consistências alimentares em lactentes no período de introdução da alimentação complementar.

## ESTRATÉGIA DE PESQUISA

Este estudo trata-se de uma revisão sistemática conduzida e relatada de acordo com o PRISMA 2020 (*Preferred Reporting Items for Systematic Reviews and Meta-Analysis Checklist* ^(17)^. O protocolo da presente revisão foi submetido e registrado no Registro Prospectivo Internacional de Revisões Sistemáticas (*International prospective register of systematic reviews* - PROSPERO) sob o número CRD42020192884^(17)^.

## CRITÉRIOS DE SELEÇÃO

O acrônimo “PECOS” foi utilizado para determinar os critérios de elegibilidade para responder a seguinte questão focada: “A presença da prematuridade impacta a transição das consistências alimentares em lactentes no período de introdução da alimentação complementar?”

População (P): Lactentes; Exposição (E): Prematuridade; Comparação (C): Recém-nascidos a termo; Desfecho (O): progressão de consistências alimentares em recém-nascidos prematuros com ou sem comparação; Tipos de estudos (S): Estudo de coorte; Caso-controle; Transversal.

Foram incluídos estudos cuja amostra foi composta por recém-nascidos com idade gestacional (IG) inferior a 37 semanas, portanto, prematuros sem comorbidades e/ou alterações orofaciais que possam interferir no processo alimentar. Os estudos poderiam ou não terem realizado comparações com recém-nascidos a termo no mesmo estudo. Os estudos também deveriam abordar a progressão das consistências alimentares no período de introdução da alimentação complementar e terem um desenho analítico (observacional). Não houve restrição quanto à etnia ou sexo, bem como o ano de publicação ou idioma.

Estudos que apresentavam as seguintes características foram excluídos:

1- Anomalias craniofaciais, síndromes genéticas, doenças neuromusculares, paralisia cerebral e/ou com disfagia
2- Crianças maiores de 24 meses
3- Que não continham crianças prematuras
4- Recém-nascidos com idade gestacional superior a 37 semanas, exceto as comparadas.
5- Que não incluíram a progressão das consistências alimentares em recém-nascidos prematuros com ou sem comparação.
6- Estudos descritivos, como cartas ao editor, comentários, relatos de casos, opiniões de especialistas, resumos de congressos, cartas, pôsteres, resenhas e livros.
7- Estudos realizados no período de internação neonatal sem seguimento.
8- Artigos com dados incompletos

Combinações de palavras e truncamentos apropriados foram selecionados e adaptados especificamente para cada banco de dados eletrônico: EMBASE, Literatura Latino-Americana e do Caribe em Ciências da Saúde (LILACS), LIVIVO, PubMed/Medline, Scopus e *Web of Science* (Apêndice A).

Pesquisas na literatura cinzenta, também, foram realizadas com o Google Scholar, Open Grey e ProQuest Dissertations & Theses. Após a busca nas bases de dados eletrônicas, foi realizada uma busca manual das referências dos estudos incluídos para evitar o risco de não incluir algum estudo relevante. As referências foram gerenciadas e os estudos duplicados foram removidos, usando o gerenciador de referências EndNote® (Thomson Reuters, Philadelphia, PA). As pesquisas foram realizadas em 10 de agosto de 2020 e atualizadas em 19 de setembro de 2022.

A seleção dos estudos foi realizada em duas fases. Na Fase 1, foi realizada a leitura dos títulos e resumos de todas as bases de dados eletrônicas. Todos os artigos que não atendiam aos critérios de elegibilidade foram excluídos nessa etapa. Na Fase 2, todos os estudos selecionados foram lidos na íntegra e os mesmos critérios de elegibilidade aplicados pelos mesmos revisores. Em ambas as fases, as leituras ocorreram de forma cegada e independente, por dois revisores (H.N.C.L.G e R.H.P). Qualquer discordância ou conflito entre os dois revisores nas fases 1 e 2 foram resolvidos por discussão até que um acordo mútuo fosse alcançado e, se não houvesse consenso, um terceiro revisor (S.T.A) foi consultado para a tomada de decisão final.

Antes de iniciar a leitura de fase 1, foi realizada a calibração dos dois revisores, através do índice de concordância de Kappa. A leitura somente foi iniciada após a obtenção de um índice > 0.7, indicando boa concordância entre revisores. A leitura em ambas as fases ocorreu pelo website Rayyan (https://rayyan.qcri.org/), garantindo, assim, o adequado cegamento dos revisores e maior transparência durante estas etapas.

## ANÁLISE DOS DADOS

Dois revisores (H.N.C.L.G e R.H.P) selecionaram e extraíram independentemente os dados dos artigos incluídos e compararam as informações extraídas. Qualquer discordância sobre os dados foi discutida entre eles e se necessário, um terceiro revisor (S.T.A) foi acionado para tomar a decisão final. Os seguintes dados foram extraídos dos artigos incluídos: autor; ano de publicação; país; objetivo do estudo, características da amostra (tamanho da amostra, idade, sexo, progressão das consistências alimentares); desenho do estudo, resultados e conclusão. Nos casos de dados omissos ou incompletos no artigo, foram realizadas três tentativas de contato via e-mail com os autores, com intervalos de uma semana, para obtenção de tais informações.

A qualidade metodológica dos estudos observacionais selecionados foi avaliada usando a *Meta-Analysis of Statistics Assessment and Review Instrument* (MASTARI). Dois revisores (H.N.C.L.G e R.H.P) realizaram a avaliação do risco de viés separadamente e julgaram os artigos incluídos, marcando cada critério de avaliação como: “alto”, quando o estudo teve pontuação “sim” menor que 49%; “moderado”, quando o estudo teve entre 50% e 69% de pontuações “sim”; e “baixo”, quando o estudo teve mais de 70% das pontuações “sim” para questões de risco de viés. Quando necessário, as discordâncias foram resolvidas por meio de discussão com um terceiro revisor (S.T.A).

## RESULTADOS

A busca nas bases de dados resultou em 3.310 estudos. Foi realizada a leitura dos títulos e resumos (Fase 1), e 3.195 estudos foram excluídos, após resolução de conflitos e dúvidas, bem como a exclusão de dez estudos duplicados. Foram selecionados 46 artigos para leitura na íntegra. A busca na literatura cinzenta foi realizada no Google Scholar, *Open Gray* e Proquest (Teses e Dissertações), identificando 78 estudos; entretanto, somente um foi selecionado. Foi realizada busca manual nas referências dos 46 estudos selecionados para a fase dois e identificaram-se três estudos adicionais. Desta forma, foram selecionados 50 estudos na fase 2 para a leitura do texto completo, sendo removidos 41 (Apêndice B), totalizando nove estudos para a síntese qualitativa (Figura 1).

**Figura 1:** Diagrama de fluxo PRISMA 2020 para novas revisões sistemáticas que incluíram pesquisas de bancos de dados, registros e outras fontes^(17)^

No que diz respeito ao desenho de estudo, os nove artigos eram observacionais, sendo três estudos transversais^(10,11,18)^, cinco coortes^(15,19-22)^ e um caso-controle^(8)^, publicados entre os anos de 2004^(8)^ e 2020^(22)^.

Dos nove estudos, cinco deles no idioma inglês^(8,15,18,19,22)^ e quatro em português^(10,11,20,21)^, sendo um da Inglaterra^(18)^, quatro do Brasil^(10,11,20,21,)^, dois da Austrália^(8,15)^, um dos Estados Unidos^(19)^ e um da Alemanha^(22)^.

O tamanho da amostra variou de 15^(18)^ a 85 recém-nascidos pré-termo^(15)^, com idades entre zero e 24 meses de idade corrigida.

Sobre a idade de introdução da alimentação complementar sete dos nove estudos^(10,11,15,19-22)^ relataram ter ocorrido de forma precoce, isto é, antes da idade recomendada de 6 meses de idade corrigida e do aparecimento de sinais de prontidão^(23).^

Outro dado descrito foi a presença de disfunção motora oral nas habilidades com copo, pequenos pedaços e sólidos, com presença de engasgos e comportamentos defensivos, isto é, foram observadas dificuldades referentes a apreensão labial em copo aberto e dificuldade na mastigação de pequenos pedaços e sólidos^(8,10,11,18-22)^.

Um estudo encontrou como resultado, uma tendência menor de recusa alimentar em bebês em aleitamento materno exclusivo até o 6º mês de idade^(11)^. O mesmo estudo identificou associação entre IG < 28 semanas, tempo de uso de alimentação enteral e parenteral com dificuldades alimentares. Em quatro^(19-22)^ estudos foi observado melhora da disfunção motora oral aos 12 meses, isto é, houve melhora nas habilidades de mastigação com alimentos sólidos e na consistência líquida em copo aberto. Em um^(19)^ destes estudos, foram acompanhados 41 RNPT, e, neste estudo, a maioria foi amamentada por mamadeira, outras duas^(20,21)^ pesquisas em que uma acompanhou 45 e outra 52, ambas utilizaram a mesma população e, por fim, um último estudo^(22)^ acompanhou 40 RNPT em que todos os estudos tratavam de acompanhamento até os 12 meses de idade corrigida.

Como método de coleta de dados, todos utilizaram questionário para dados demográficos e de história neonatal, para a avaliação das habilidades motoras orais o protocolo mais utilizado foi o *Schedule for Oral Motor Assessment (SOMA),* utilizado em três dos nove estudos^(18,20,21)^. Os demais estudos utilizaram diferentes protocolos como: Checklist Sociedade Brasileira de Pediatria^(11)^, Protocolo de Avaliação Clínica da Disfagia Pediátrica (PAD-PED)^(11)^, *The child feeding skills checklist* ^(19)^, *Neonatal Oral Motor Assessment Scale (NOMAS)* ^(22)^, *Observation List Spoon Feeding (OSF)* ^(22)^, *Mastication Observation and Evaluation Instrument (MOE)* ^(22)^, *Royal Children's Hospital Oral Sensitivity Checklist (OSC)* ^(8)^ *e Pré-speech Assessment Scale (PSAS)* ^(8)^, já em outro^(15)^ não foi utilizado nenhum protocolo específico para avaliação motora oral, somente um questionário estruturado elaborado pelos autores.

Dentre os três estudos observacionais transversais incluídos, um^(18)^ apresentou baixo risco de viés e dois^(10,11)^ tiveram um risco moderado. Dentre estes estudos, os três^(10,11,18)^ foram avaliados negativamente por não apresentarem uma amostra aleatória. Em dois^(10,11)^ estudos, os fatores de interferência não foram reconhecidos e as abordagens para lidar com eles foram mencionadas ou não estavam explícitas, além disso, os resultados dos participantes que se retiraram não foram detalhados e considerados na avaliação. Em cinco estudos observacionais de Coorte foi verificado um^(15)^ com risco moderado, pois os resultados não foram avaliados por meio de critérios objetivos, os resultados das pessoas que se retiraram não foram descritos e incluídos na análise e os resultados não foram medidos de forma confiável. Os outros quatro^(19-22)^ apresentaram baixo risco de viés, o único estudo caso-controle^(8)^ incluído apresentou baixo risco de viés (Apêndice C).

Os dados extraídos de cada estudo incluído, podem ser visualizados no Quadro 1. Nos estudos selecionados foi observada uma tendência de introdução de alimentos sólidos mais tarde, considerando a idade cronológica entre os prematuros quando comparados aos a termo, entretanto, quando considerada a idade corrigida, os alimentos sólidos foram introduzidos mais precocemente nos prematuros^(10,11,15)^.

**Quadro 1:** Características dos estudos incluídos (n = 9)

Autor (País)	Tamanho da amostra	Idade, sexo	Desenho do estudo	Objetivo	Resultados e Conclusão
Brusco e Delgado^(10)^ (Brasil)	32 RNPT	Média de 4 meses 27 dias e IC 1 mês 27 dias	Transversal	Caracterizar o desenvolvimento da alimentação de RNPT, entre três e 12 meses, verificar o tipo de aleitamento, época da IA, hábitos orais deletérios, orientações recebidas, dificuldades alimentares e perfil sociodemográfico.	Observada oferta de líquidos precocemente. O pastoso iniciado em idade adequada, mas sólidos de forma precoce, 17 relataram dificuldade alimentar. Houve associação entre recusa e IG e baixo peso, bem como hipotonia de lábio, língua e bochecha com a IG.
		Min 90 dias e máx 10 meses 29 dias			
		21 masculino			
		11 feminino			
Buswell et al.^(18)^ (Inglaterra)	15 RNPT	10 meses IC	Transversal	Determinar presença de DMO durante a alimentação na primeira infância e se fatores neonatais associados a dificuldades de alimentação são preditores de DMO	3 crianças apresentaram escore limite ou indicativo de DMO, 1 criança com DMO para todas as consistências, 2 com DMO para todas exceto o sólido, e 3 não se alimentavam com sólido.
		9 masculino			12 bebês aceitavam todas as consistências e não houve relação das variáveis neonatais com a pontuação do SOMA.
		6 feminino			
Cleary et al.^(15)^ (Austrália)	RNPT 85	21 semanas RNPT	Coorte prospectivo	Determinar a idade da introdução de alimentos sólidos em RNPT, comparados a RNT e os fatores associados.	Tiveram introdução precoce de sólidos em RNPT.
	RNT 65	19 semanas RNT			Tipo de aleitamento e peso de nascimento não tiveram associação com idade de introdução de sólidos.
		56 masculino			Em RNT, menor escolaridade e menor idade materna teve associação com introdução de sólidos precocemente.
		29 feminino			A DMO não foi avaliada neste estudo.
Dodrill et al.^(8)^ (Austrália)	20 RNPT	Média de 11-17 meses IC	Caso Controle	Verificar diferenças entre a sensibilidade oral e o desenvolvimento alimentar de RNPT e RNT e examinar diferenças na sensibilidade oral e o desenvolvimento alimentar de RNPT e RNT que receberam períodos mais curtos de alimentação via SNG e maior duração da alimentação por SNG	Foram observadas diferenças entre RNPT e RNT, nos comportamentos sugestivos de sensibilidade oral alterada (p = 0,000), e os RNPT apresentaram mais protrusão de língua (p = 0,010) e escape ao deglutir sólidos (p = 0.006).
	10 RNT	Feminino 6 RNT e 11 RNP			
		Masculino 4 RNT e 9 RNPT			
Ferreira^(20)^ (Brasil)	45 RNPT	M1 32,9 semanas	Coorte prospectivo	Verificar o estado nutricional e o desenvolvimento das habilidades motoras orais em RNPT durante o primeiro ano de vida e as possíveis associações entre eles.	Aos 4 meses foi observada disfunção motora oral em 78% para o purê, aos 6 meses diminuiu para 41%.
		M2 37,2s			Na consistência semissólida aos 6m foi observado DMO em 9% e nenhum para líquidos.
		M3 4,1 meses			Aos 9 meses, 14% apresentaram DMO para líquido em copo plástico. Aos 12m, a DMO para líquido foi em 13% e 9% para sólido.
		M4 6m			
		M5 8,9m			
		M6 11,9m			
		24 feminino / 21 masculino			
Hübl et al.^(22)^ (Alemanha)	40 RNPT	6/9/12/24 meses	Coorte prospectivo	Examinar DMO precoce e relacionar com dificuldades na alimentação com semissólidos e sólido	A introdução alimentar foi iniciada com purê por todos e com idade inferior a recomendada.
		21 feminino			Metade dos lactentes receberam alimentos semissólidos e sólidos antes da idade recomendada.
		19 masculino			Com 12 meses (idade pós-menstrual), 6 lactentes não estavam recebendo sólidos, devido a engasgos e medo dos pais. Na avaliação de alimentação por colher aos 6m, 10% alcançaram pontuação total, 9m, 65,8% e aos 12m, 71,8%. Foi observado melhora na mastigação de 9 a 12 m e de 9 a 24m.
Pridham et al.^(19)^ (Estados Unidos)	41 RNPT	Média min 40,2 max 375,1 dias	Coorte prospectivo	Examinar a contribuição independente e interativa das condições biológicas e maternas de alimentação infantil para desempenho da habilidade de alimentação	Somente 51% apresentaram um sinal de prontidão para IA.
		1 av. 22 masculino e feminino 22			Aos 8 meses, 60% apresentaram habilidades com copo e coordenação para alimentos granulados.
		2 av. 19 masculino e feminino 16			Com 12 meses, 70% apresentaram habilidades para comer todas as consistências.
		3 av. 21 masculino e feminino 17			
		4 av. 21 masculino e feminino 20			
Steinberg et al.^(11)^ (Brasil)	62 RNPT	Média 13,5 meses IC	Transversal	Investigar se existe associação entre disfunção motora oral e dificuldade alimentar durante o processo de introdução de alimentação complementar em crianças nascidas pré-termo	27 mães referiram dificuldade em alimentar seus filhos. E após a aplicação do checklist, este número aumentou, sendo que 43 delas referiram que seu filho tinha alguma dificuldade.
		36 feminino			Crianças em aleitamento materno exclusivo até 6 meses, foi observado menor tendência de recusa alimentar.
		26 masculino			O comportamento defensivo mais frequente foi a recusa em abrir a boca. Não foi observada associação em dificuldade motora oral e dificuldade alimentar.
					Entretanto, houve associação entre comida liquidificada, IG, tempo de SNE, com dificuldade alimentar.
Yamamoto^(21)^ (Brasil)	52 RNPT	4/6/9/12 meses	Coorte prospectivo	Investigar se existe associação entre disfunção motora oral e dificuldade alimentar durante o processo de introdução de alimentação complementar em crianças nascidas pré-termo	Aos 4 meses, a maioria apresentou disfunção motora oral, não foi observado associação entre resultado da SOMA para purê com idade gestacional. Aos 6 meses, 65,6% apresentaram função motora oral normal para purê, e para o semissólido, 97% apresentaram normalidade. Aos 9m e aos 12m, mais de 85% apresentaram função motora oral normal para todas as consistências.
		28 feminino			
		24 masculino			

Legenda: RNPT = Recém-nascido prematuro; RNT = Recém-nascido a termo; IC = Idade Corrigida; IG = Idade Gestacional; DMO = Disfunção Motora Oral; IA = Introdução Alimentar; SNG = Sonda Nasogástrica

Fonte: Os autores (2021)

Houve, também, relação entre intervenções orais invasivas e dificuldade alimentar com p < 0,05 em todas as habilidades avaliadas^(11)^.

Na associação entre idade gestacional e dificuldades alimentares foi observada significância estatística, demonstrando que quanto menor a idade gestacional maior frequência de comportamentos de dificuldade alimentar^(8,10,11)^. A disfunção motora oral relacionada à idade gestacional foi descrita em dois estudos^(10,21)^. Também, foi encontrada associação entre disfunção motora oral, idade gestacional e a consistência sólida^(21)^, sendo observada esta mesma relação em outro estudo^(18)^.

No que diz respeito à progressão das consistências, na comparação entre avaliações aos 4 meses e 6 meses de idade corrigida, é possível verificar que a frequência de disfunção motora oral para a consistência purê diminui significativamente^(20)^. Também, foi verificado melhora nas habilidades quando comparadas as avaliações aos 9 e 12 meses, e aos 9 meses e 24 meses^(19,22)^.

Em nenhum dos estudos há citação ou inferência de que os participantes passaram por algum tipo de intervenção, seja orientativa ou de reabilitação.

Devido à heterogeneidade metodológica existente entre os estudos e a diversidade de protocolos de avaliação utilizados, não foi possível realizar a síntese quantitativa. O único protocolo utilizado em pelo menos três estudos foi o protocolo *Schedule for Oral Motor Assessment* (SOMA), contudo, destes três estudos, dois compartilhavam da mesma amostra, não sendo indicada, desta forma, a realização da síntese quantitativa para não haver duplicação dos dados.

Estima-se que nasçam anualmente mais de 15 milhões de prematuros no mundo, e, em consequência da qualidade tecnológica de assistência a esses recém-nascidos há, também, um aumento de sobrevida, trazendo em longo prazo custos, em decorrência de atrasos de desenvolvimento, dificuldades alimentares e outras comorbidades^(24)^.

O Ministério da Saúde do Brasil e a Sociedade Brasileira de Pediatria recomendam que a introdução de alimentação complementar seja iniciada aos 6 meses de idade no RNT^(25)^ e no RNPT, a introdução é recomendada aos seis meses de idade corrigida^(23)^.

Além da idade, deve-se considerar o neurodesenvolvimento no bebê, bem como idade corrigida, para os prematuros^(26)^. Entretanto tal prática não foi observada nos estudos desta revisão, na qual se pode observar que a introdução da alimentação complementar ocorreu de forma precoce em sete dos nove estudos, por volta dos quatro meses de idade corrigida^(10,11,15,19-22)^. Foi observada, também, a introdução de alimentação complementar mais tarde nos prematuros em relação aos a termo com diferença significativa (p < 0,02)^(15)^, porém isto sem considerar a idade corrigida. Quando há a correção da idade, observa-se que os alimentos sólidos são introduzidos mais precocemente nos prematuros, mantendo, ainda, diferença significativa (p<0,001). Este dado é relevante, pois estudos que comparam a introdução de alimentos aos quatro meses e aos seis meses observam um aumento no risco de infecções quando a introdução de alimentação complementar acontece aos 4 meses^(24)^, além de risco aumentado de alergias alimentares e obesidade^(27)^.

Vale ressaltar ainda sobre este assunto, que na literatura não há consenso nas recomendações de quando a introdução da alimentação complementar deve iniciar em lactentes prematuros. O Comitê de Nutrição da Sociedade Europeia de Gastroenterologia, Hepatologia e Nutrição Pediátrica (ESPGHAN), assim como a Organização Mundial da Saúde, recomendam fortemente a oferta de leite materno aos prematuros, enfatizando a importância do apoio e acompanhamento da díade mãe/bebê para qualificar e expandir o tempo de aleitamento materno. Além disso, levando-se em conta que esta população apresenta risco nutricional aumentado, seria indicado uma abordagem individualizada de acordo com a capacidade neurológica e o estado nutricional do lactente para uma prática na introdução da alimentação complementar em prematuros mais assertiva, especialmente na ausência de diretrizes baseadas em evidências^(24)^.

Na presente revisão, um estudo^(11)^ demonstrou a relação entre intervenções orais invasivas e dificuldade alimentar com significância estatística em todas as habilidades avaliadas, corroborando com outros estudos que relatam risco de dificuldades alimentares decorrentes de intervenções as quais foram submetidas, ainda, no ambiente hospitalar em unidade de terapia intensiva, em bebês prematuros^(28)^. Tais intervenções promovem estímulos orais que provocam um déficit de experiência sensorial adequada nos primeiros meses, acarretando em reflexos orais exacerbados e/ou suprimidos^(29)^. Vale ressaltar que em se tratando da alimentação complementar nos primeiros anos de vida, há de se considerar inúmeros fatores que influenciam tanto para o início quanto para a progressão das consistências, sendo indicado um acompanhamento com protocolos validados, prevendo os vários vieses interferentes para melhor compreensão de tais relações.

As habilidades motoras finas e grossas, que são adquiridas com o desenvolvimento neuropsicomotor adequado, fazem parte da constituição das habilidades alimentares^(30)^. O atraso na aquisição destas habilidades é observado de forma frequente em RNPT. Apenas um estudo não observou relação entre idade gestacional e DMO^(18)^ e trata-se de um estudo com uma amostra pequena de 36 lactentes, com idade gestacional < 37 semanas ao nascimento. Já três outros estudos apontam que quanto menor a idade gestacional, mais frequente são os comportamentos de dificuldades alimentares, com significância estatística^(8,10,11)^.

A DMO, propriamente dita, teve relação com idade gestacional em dois estudos^(10,21)^. Em ambos, a população estudada foi de prematuros com avaliação fonoaudiológica, entretanto o primeiro realizou um acompanhamento de RNPT extremos e tardios, desde o nascimento até os 12 meses, e o segundo, realizou estudo transversal com RNPT tardios. Porém, neste último, a amostragem não foi feita de forma aleatória.

Outro estudo^(12)^ que aplicou um questionário aos pais de crianças aos 2 anos, constatou que 14,9% dos lactentes prematuros tardios e moderados tiveram dificuldades alimentares aos dois anos em comparação aos a termo, com 9,5%, o que representa um aumento de 57% de dificuldades alimentares entre os bebês prematuros, dentre eles, problemas motores orais.

No que diz respeito à progressão das consistências, foi observado melhora nas habilidades orais com a consistência purê entre os quatro e os seis meses de idade corrigida^(20)^. Também, foi verificado melhora nas habilidades quando comparado entre nove meses e 12 meses, e nove meses e 24 meses com p = 0,001^(19,22)^. Estes dados concordam com os dados de uma revisão na qual foi observada que a maturidade da habilidade alimentar ocorre à medida que acontecem mudanças anatômicas durante o crescimento, desenvolvimento neuropsicomotor, bem como as experiências frente aos tipos de alimentos, texturas e sua apresentação nos primeiros anos de vida^(7)^. Este período proporciona ao lactente experiências com novas texturas e sabores alimentares, contribuindo na modulação e conexões cerebrais responsáveis pelo controle da ingesta com resultados em longo prazo^(13)^.

Entretanto, os estudos não citam se houve ou não intervenção terapêutica fonoaudiológica, o que pode influenciar na frequência das dificuldades, uma vez que um acompanhamento especializado, orientações e até mesmo intervenções frente a dificuldades iniciais na introdução da alimentação complementar, podem prevenir que esta população venha apresentar atrasos.

Também, importante considerar que há uma heterogeneidade nos protocolos utilizados e uma falta de padronização dos instrumentos e do método utilizado entre os estudos para a avaliação de problemas na progressão das consistências no período de introdução da alimentação complementar, o que demonstra que não existe ainda um instrumento ideal para avaliação das habilidades alimentares nesta faixa etária, e há uma necessidade de consenso que padronize uma avaliação mais assertiva.

## CONCLUSÃO

Não foi observada relação entre a prematuridade e dificuldades na progressão das consistências no período da introdução das consistências alimentares na maioria dos estudos, somente três deles demonstraram que existe uma relação com dificuldade alimentar, caracterizada por recusa alimentar, recusa em abrir a boca, vômito e sinais defensivos durante a oferta.

Alguns estudos apontaram para uma melhora nas habilidades orais à medida que os RNPT crescem e apresentam sinais de prontidão, com consequente melhora nas habilidades para as consistências semissólida e sólida de 12 a 24 meses.

Os estudos selecionados apresentaram heterogeneidade metodológica e diversidade dos protocolos utilizados.

Vale ressaltar a importância na padronização de instrumentos de *screening* e mais estudos da avaliação de DMO em RNPT e comparação com RNT para verificar de forma mais robusta possíveis dificuldades e a necessidade de instituir o acompanhamento e intervenção precoce a fim de prevenir dificuldades alimentares, nutricionais e do desenvolvimento neuropsicomotor em RNPT.

## Apêndice A Estratégia de pesquisa em banco de dados

**Table:**

Database	Search (April, 24^th^, 2020)
Lilacs	(“premature birth” OR “Very preterm infant” OR “Premature Births” OR “Preterm Birth” OR “Preterm Births” OR “Premature Infant” OR “Very preterm birth” OR “Preterm Infants” OR “Preterm Infant” OR “Premature Infants” OR “nascimento prematuro” OR “Bebê muito prematuro” OR “Nascimentos prematuros” OR “Bebê prematuro” OR “Nascimento muito prematuro” OR “Bebês prematuros” OR “nacimiento prematuro” OR “bebé muy prematuro” OR “nacimientos prematuros” OR “bebé prematuro” OR “nacimiento muy prematuro” OR “bebés prematuros”) AND (“Feeding Behavior” OR “Feeding Behaviors” OR “Eating Behavior” OR “Eating Behaviors” OR “oral feeding progression” OR “solid food introduction” OR “past food introduction” OR “Comportamento alimentar” OR “Comportamentos alimentares” OR “progressão da alimentação oral” OR “introdução de alimento sólido” OR “Comportamiento alimenticio” OR “Comportamientos alimentícios” OR “progresión de la alimentación oral” OR “introducción de alimentos sólidos”)
PubMed	1. (“premature birth”[MeSH Terms] OR “premature birth”[All Fields] OR “Very preterm infant”[All Fields] OR “Premature Births”[All Fields] OR “Preterm Birth”[All Fields] OR “Preterm Births”[All Fields] OR “Premature Infant”[All Fields] OR “Very preterm birth”[All Fields] OR “Preterm Infants”[All Fields] OR “Preterm Infant”[All Fields] OR “Premature Infants”[All Fields]) 2. (“feeding behavior”[MeSH Terms] OR “feeding behavior”[All Fields] OR “Feeding Behaviors”[All Fields] OR “Eating Behavior”[All Fields] OR “Eating Behaviors”[All Fields] OR “oral feeding progression”[All Fields] OR “solid food introduction”[All Fields]) 3. #1 AND #2
SCOPUS	TITLE-ABS-KEY(“premature birth” OR “Very preterm infant” OR “Premature Births” OR “Preterm Birth” OR “Preterm Births” OR “Premature Infant” OR “Very preterm birth” OR “Preterm Infants” OR “Preterm Infant” OR “Premature Infants”) AND TITLE-ABS-KEY(“Feeding Behavior” OR “Feeding Behaviors” OR “Eating Behavior” OR “Eating Behaviors” OR “oral feeding progression” OR “solid food introduction” OR “past food introduction”)
Web of Science	8. TS=(“premature birth” OR “Very preterm infant” OR “Premature Births” OR “Preterm Birth” OR “Preterm Births” OR “Premature Infant” OR “Very preterm birth” OR “Preterm Infants” OR “Preterm Infant” OR “Premature Infants”) 9. TS=(“Feeding Behavior” OR “Feeding Behaviors” OR “Eating Behavior” OR “Eating Behaviors” OR “oral feeding progression” OR “solid food introduction” OR “past food introduction”) 10. #1 AND #2
LIVIVO	TI=(“premature birth” OR “Very preterm infant” OR “Premature Births” OR “Preterm Birth” OR “Preterm Births” OR “Premature Infant” OR “Very preterm birth” OR “Preterm Infants” OR “Preterm Infant” OR “Premature Infants”) AND TI=(“Feeding Behavior” OR “Feeding Behaviors” OR “Eating Behavior” OR “Eating Behaviors” OR “oral feeding progression” OR “solid food introduction” OR “past food introduction”)
Embase	('premature birth'/exp OR 'premature birth' OR 'very preterm infant'/exp OR 'very preterm infant' OR 'premature births' OR 'preterm birth'/exp OR 'preterm birth' OR 'preterm births' OR 'premature infant'/exp OR 'premature infant' OR 'very preterm birth'/exp OR 'very preterm birth' OR 'preterm infants' OR 'preterm infant'/exp OR 'preterm infant' OR 'premature infants') AND ('feeding behavior'/exp OR 'feeding behavior' OR 'feeding behaviors' OR 'eating behavior'/exp OR 'eating behavior' OR 'eating behaviors' OR 'oral feeding progression' OR 'solid food introduction' OR 'past food introduction')
Google Scholar	“premature birth” AND “feeding behavior”
Open Grey	“premature birth”
ProQuest	(“premature birth” OR “Very preterm infant” OR “Premature Births” OR “Preterm Birth” OR “Preterm Births” OR “Premature Infant” OR “Very preterm birth” OR “Preterm Infants” OR “Preterm Infant” OR “Premature Infants”) AND (“Feeding Behavior” OR “Feeding Behaviors” OR “Eating Behavior” OR “Eating Behaviors” OR “oral feeding progression” OR “solid food introduction” OR “past food introduction”)

Fonte: Os autores (2021)

## Apêndice B Artigos excluídos e as razões de exclusão (n= 41)

**Table:**

Autor, ano	Razões de exclusão
A. Kirk, S. Alder, J. King; 2006	11
I. Adams-Chapman, C.M. Bann, Y.E. Vaucher, MD, B.J. Stoll; 2013	5
R. Barachetti, E. Villa, M. Barbarini; 2017	10
E. N. Bezze, M.L. Giannì, P. Sannino, C. Esposito, L. Plevani, S. Muscolo, P. Roggero, F. Mosca; 2017	10
J.V. Browne, E.S. Ross; 2011	10
A.N. Coşkun, P.Z. Akkuş, E.I. Bahadur, H.T.Çelik, E.N. Özmert, 2019	10
T.L. Crapnell, C.E. Rogers, J.J. Neil, T.E. Inder, L.J. Woodward, R.G. Pineda, 2013	9
Crapnell, T.L., Woodward, L.J., Rogers, C.E., Inder, T.E., Pineda, R.G., 2015	9
Delaney, A.L., Arvedson, J.C., 2008	10
DeMauro SB, Patel PR, Medoff-Cooper B, Posencheg M, Abbasi S., 2011	9
S.L. den Boer, J.A. Schipper, 2013	9
P. Dodrill,; T. Donovan; 2014	10
N.D. Embleton, M. Fewtrell, 2017	10
S. Fanaro, G. Borsari, V. Vigi, 2007	9
Giannì M.L., Bezze E, Colombo L, Rossetti C, Pesenti N, Roggero P, Sannino P, Muscolo S, Plevani L, Mosca F., 2018	9
Howe T.H., Sheu C.F., Wang T.N., 2019	9
S. Johnson, R. Matthews, E.S. Draper, D.J. Field, B.N. Manktelow, N.Marlow, L.K. Smith, E.M. Boyle, 2016	9
Kennedy C., Lipsitt L.P., 1993	10
King, C., 2009	10
Méio M.D.B.B., Villela L.D., Gomes Júnior S.C.D.S., Tovar C.M., Moreira M.E.L., 2018	9
Menezes LVP, Steinberg C, Nóbrega AC., 2017	9
Migraine A, Nicklaus S, Parnet P, Lange C, Monnery-Patris S, Des Robert C, Darmaun D, Flamant C, Amarger V, Rozé JC., 2013	9
Navarro, L.; Antunes, H.; 2019	7
Norris, F., Larkin, M., Williams, C. Hampton, S.M. Morgan, J.B., 2002	9
O'Grady, R. S.; 1971	10
Palmer D.J., Makrides M., 2012	10
Patra, K., Greene, M.M., 2019	9
Philip, A. K.; Vijay Kumar, K. V., 2015	9
Pineda, R. G., 2016	10
Pridham, K.; Saxe, R.; Limbo, R.; 2004	10
Rodriguez J, Affuso O, Azuero A, Downs CA, Turner-Henson A, Rice M., 2018	9
Ross, E. S.; Browne, J. V.; 2002	10
Silberstein, D.; Feldman, R.; Gardner, J. M.; Karmel, B. Z.; Kuint, J.; Geva, R.; 2009	9
Törölä H, Lehtihalmes M, Yliherva A, Olsén P. 2012	10
van Dijk M, Bruinsma E, Hauser MP, 2016	9
Yrjänä JMS, Koski T, Törölä H, Valkama M, Kulmala P. 2018	9
Zielinska MA, Rust P, Masztalerz-Kozubek D, Bichler J, Hamułka J. 2019	9
Cerro N.,Zeunert S., Simmer KN, Daniels A., 2002	9
Pridham K., Brown R., Clark R., Limbo RK., 2005	9
Fewtrell M., Lucas A., Morgan JB., 2018	10
Chung, J.; Lee, J.; Spinazzola, R.; Rosen, L.; Milanaik, R.; 2014	9

Legenda: 1- Pacientes com anomalias craniofaciais; 2- Pacientes com síndromes genéticas; 3- Pacientes com doenças neuromusculares; 4- Pacientes com paralisia cerebral; 5- Pacientes com Disfagia; 6- Estudos com crianças com idade maior de 24 meses; 7- Estudos que não incluam crianças prematuras; 8- Recém-nascidos com idade gestacional maior de 37 semanas; 9- Estudos que não incluem a progressão das consistências alimentares em recém-nascidos prematuros com ou sem comparação; 10- Estudos descritivos, como cartas ao editor, comentários, relatórios de casos, opiniões de especialistas, resumos de conferências, cartas, pôsteres, resumos de conferências; 11- Estudos realizados no período de internação neonatal

Referências

1 - Kirk A, Alder S, King J. 109 Risk factors for poor oral feeding progression in premature infants. Journal of Investigative Medicine 2006;54:S98.

2 - Adams-Chapman I, Bann CM, Vaucher YE, Stoll BJ; Eunice Kennedy Shriver National Institute of Child Health and Human Development Neonatal Research Network. Association between feeding difficulties and language delay in preterm infants using Bayley Scales of Infant Development-Third Edition. J Pediatr. 2013;163(3):680-5.e53.

3 - Barachetti R, Villa E, Barbarini M. Weaning and complementary feeding in preterm infants: management, timing and health outcome. Pediatr Med Chir. 2017 Dec 22;39(4):181. doi: 10.4081/pmc.2017.181. PMID: 29502384.

4 - Bezze EN Giannì ML, Sannino P, Esposito C, Plevani L, Muscolo S. *et al*. The assessment of oral feeding skills in preterm infants. Pediatria Medica e Chirurgica. 2017; 39(1): 17.

5 - Browne JV, Ross ES. Eating as a Neurodevelopmental Process for High-Risk Newborns. Clinics in Perinatology. 2011; 38 (4): 731-743.

6 – Coşkun AN, Akkuş PZ., Bahadur EI, Çelik HT, Özmert EN, Early feeding problems and associated factors in premature infants. Archives of Disease in Childhood. 2019; 104 (0): A208-A209.

7 – Crapnell, TL., Rogers, CE., Neil, JJ., Inder, TE., Woodward, L.J., Pineda, R.G. Factors associated with feeding difficulties in the very preterm infant. Acta Paediatrica, 2013 Dec;102(12):e539-45.

8 - Crapnell TL, Woodward LJ, Rogers CE, Inder TE, Pineda RG. Neurodevelopmental Profile, Growth, and Psychosocial Environment of Preterm Infants with Difficult Feeding Behavior at Age 2 Years. J Pediatr. 2015 Dec;167(6):1347-53.

9 - Delaney AL, Arvedson JC. Development of swallowing and feeding: prenatal through first year of life. Dev Disabil Res Rev. 2008;14(2):105-17. doi: 10.1002/ddrr.16. PMID: 18646020.

10 - DeMauro SB, Patel PR, Medoff-Cooper B, Posencheg M, Abbasi S. Postdischarge feeding patterns in early- and late-preterm infants. Clin Pediatr (Phila). 2011 Oct;50(10):957-62.

11 - den Boer SL, Schipper JA. Feeding and drinking skills in preterm and low birth weight infants compared to full term infants at a corrected age of nine months. Early Hum Dev. 2013 Jun;89(6):445-7. doi: 10.1016/j.earlhumdev.2012.12.004. Epub 2012 Dec 27.

12 - Dodrill P, Donovan T. Feeding skills and dietary intake of preterm infants at 12 months corrected age. Dysphagia. 2014; 29 (6): 758.

13 - Embleton ND, Fewtrell M. Complementary feeding in preterm infants. The Lancet. 2017; 5(5): 470-e471.

14 - Fanaro S, Borsari G, Vigi V. Complementary feeding practices in preterm infants: an observational study in a cohort of Italian infants. J Pediatr Gastroenterol Nutr. 2007 Dec;45 Suppl 3:S210-4.

15 - Giannì ML, Bezze E, Colombo L, Rossetti C, Pesenti N, Roggero P, Sannino P, Muscolo S, Plevani L, Mosca F. Complementary Feeding Practices in a Cohort of Italian Late Preterm Infants. Nutrients. 2018 Dec 2;10(12):1861.

16 - Howe TH, Sheu CF, Wang TN. Feeding Patterns and Parental Perceptions of Feeding Issues of Preterm Infants in the First 2 Years of Life. Am J Occup Ther. 2019 Mar/Apr;73(2):7302205030p1-7302205030p10.

17 – Johnson S, Matthews R, Draper ES, Field DJ, Manktelow BN, Marlow N. *et al*. Eating difficulties in children born late and moderately preterm at 2 y of age: a prospective population-based cohort study–, The American Journal of Clinical Nutrition. 2016; 103(2): 406–414

18 - Kennedy C, Lipsitt LP. Temporal characteristics of non-oral feedings and chronic feeding problems in premature infants. J Perinat Neonatal Nurs. 1993 Dec;7(3):77-89.

19 - King, C. An evidence based guide to weaning preterm infants. Symposium: Nutrition. Paediatrics and Child Health.2009; 19(9): 405-414.

20 - Méio MDBB, Villela LD, Gomes Júnior SCDS, Tovar CM, Moreira MEL. Breastfeeding of preterm newborn infants following hospital discharge: follow-up during the first year of life. Cien Saude Colet. 2018; 23 (7): 2403-2412.

21 – Menezes LVP, Steinberg C, Nóbrega AC. Complementary feeding in infants born prematurely. Codas. 2018; 30(6): e20170157.

22 - Migraine A, Nicklaus S, Parnet P, Lange C, Monnery-Patris S, Des Robert C, Darmaun D, Flamant C, Amarger V, Rozé JC. Effect of preterm birth and birth weight on eating behavior at 2 y of age. Am J Clin Nutr. 2013 Jun;97(6):1270-7.

23 - Navarro L, Antunes H, Baby-led weaning: A trend in complementary feeding? A population based cross-sectional study. Journal of Pediatric Gastroenterology and Nutrition. 2019; 68 (0): 352, 2019.

24 - Norris F, Larkin M, Williams C, Hampton SM, Morgan JB. Factors affecting the introduction of complementary foods in the preterm infant. Eur J Clin Nutr. 2002; 56: 448–454.

25 - O'Grady RS. Feeding behavior i infants. Am J Nurs. 1971; 71 (4): 736-9.

26 - Palmer DJ, Makrides M. Introducing solid foods to preterm infants in developed countries. Ann Nutr Metab. 2012; 60 (2):31-8.

27 - Patra K, Greene MM. Impact of feeding difficulties in the NICU on neurodevelopmental outcomes at 8 and 20 months corrected age in extremely low gestational age infants. J Perinatol. 2019 Sep;39(9):1241-1248.

28 - Philip AK, Vijay Kumar KV. Comparison of feeding behaviours in term infants and preterm infants (30 to 34 weeks) at six months corrected age. Journal of Nepal Paediatric Society. 2015; 35 (2): 202-205.

29 - Pineda RG. Feeding: an important, complex skill that impacts nutritional, social, motor and sensory experiences. Acta Paediatrica, International Journal of Paediatrics. 2016; 105 (10): e458.

30 - Pridham K, Saxe R, Limbo R, Feeding issues for mothers of very low-birth-weight, premature infants through the first year. J Perinat Neonatal Nurs. 2004; 18 (2): 161-9.

31 - Rodriguez J, Affuso O, Azuero A, Downs CA, Turner-Henson A, Rice M. Infant Feeding Practices and Weight Gain in Toddlers Born Very Preterm: A Pilot Study. J Pediatr Nurs. 2018; 43: 29-35.

32 - Ross ES, Browne JV. Developmental progression of feeding skills: an approach to supporting feeding in preterm infants. Semin Neonatol. 2002; 7 (6): 469-75.

33 - Silberstein D, Feldman R, Gardner JM, Karmel BZ, Kuint J, Geva R. The mother-infant feeding relationship across the first year and the development of feeding difficulties in low-risk premature infants. Infancy. 2009; 14 (5): 501-525.

34 - Törölä H, Lehtihalmes M, Yliherva A, Olsén P. Feeding skill milestones of preterm infants born with extremely low birth weight (ELBW). Infant Behav Dev. 2012 Apr;35(2):187-94.

35 - van Dijk M, Bruinsma E, Hauser MP. The relation between child feeding problems as measured by parental report and mealtime behavior observation: A pilot study. Appetite. 2016 Apr 1;99:262-267.

36 - Yrjänä JMS, Koski T, Törölä H, Valkama M, Kulmala P. Very early introduction of semisolid foods in preterm infants does not increase food allergies or atopic dermatitis. Ann Allergy Asthma Immunol. 2018 Sep;121(3):353-359.

37 - Zielinska MA, Rust P, Masztalerz-Kozubek D, Bichler J, Hamułka J. Factors Influencing the Age of Complementary Feeding-A Cross-Sectional Study from Two European Countries. Int J Environ Res Public Health. 2019 Oct 9;16(20):3799.

38 – Cerro N, Zeunert S, Simmer KN, Daniels LA. Eating behaviour of children 1.5-3.5 years born preterm: parents' perceptions. J Paediatr Child Health. 2002 Feb;38(1):72-8. doi: 10.1046/j.1440-1754.2002.00728.x. PMID: 11869405.

39 - Pridham K, Brown R, Clark R, Limbo RK, Schroeder M, Henriques J, Bohne E. Effect of guided participation on feeding competencies of mothers and their premature infants. Res Nurs Health. 2005 Jun;28(3):252-67. doi: 10.1002/nur.20073. PMID: 15884024.

40 - Fewtrell MS, Lucas A, Morgan JB. Fatores associados ao desmame em bebês nascidos a termo e pré-termo. Arch Dis Child Fetal Neonatal Ed. 2003; 88 (4): F296-F301. doi: 10.1136 / fn.88.4.f296

41 - Chung J, Lee J, Spinazzola R, Rosen L, Milanaik R. Parental perception of premature infant growth and feeding behaviors: use of gestation-adjusted age and assessing for developmental readiness during solid food introduction. Clin Pediatr (Phila). 2014 Nov;53(13):1271-7. doi: 10.1177/0009922814540039. Epub 2014 Jun 24. PMID: 24961782.

## Apêndice C Risco de viés para os estudos incluídos na análise qualitativa e quantitativa, avaliada pela ferramenta de “Meta-Análise de Instrumento de Avaliação e Revisão Estatística” (MASTARI). o risco de viés foi categorizado como alto, quando o estudo atinge até 49% de pontuação “sim”; moderado, quando o estudo atingiu 50% a 69% de pontuação “Sim” e baixo, quando o estudo atingiu mais de 70% de pontuação “Sim”

**3.1 -** Estudos incluídos na análise qualitativa

**Table:** A – Transversal.

Question					
	Buswell et al.^(18)^	Steinberg et al.^(11)^	Brusco and Delgado^(10)^		
1. Was the study based on a random or pseudorandom sample?	N	N	N		
2. Were the criteria for inclusion in the sample clearly defined?	Y	Y	Y		
3. Were confounding factors identified and strategies to deal with them stated?	Y	N	U		
4. Were outcomes assessed using objective criteria?	Y	Y	Y		
5. If comparisons are being made, was there sufficient description of the groups?	NA	NA	NA		
6. Was the follow up carried out over a sufficient time period?	Y	U	Y		
7. Were the outcomes of people who withdrew described and included in the analysis?	Y	N	N		
8. Were the outcomes measured in a reliable way?	Y	Y	U		
9. Was an appropriate statistical analysis used?	Y	Y	Y		
% yes/risk	87.5% Low	50% Moderate	50% Moderate		

Caption = Y = Yes; N = No; U = Unclear; NA = Not applicable

**Table:** B - Estudo de coorte/Estudo Caso-controle.

Question	Cleary et al.^(15)^	Pridham et al.^(19)^	Hübl et al.^(22)^	Ferreira^(20)^	Yamamoto^(21)^	Dodrill et al.^(8)^
1. Was the sample representative of patients in the population as a whole?	Y	Y	Y	Y	Y	N
2. Were the patients at a similar point in the course of their condition/illness?	Y	Y	Y	Y	Y	Y
3.Had bias been minimized in relation to selection of cases and of controls?	Y	Y	NA	Y	Y	Y
4. Were confounding factors identified and strategies to deal with them stated?	Y	Y	Y	Y	Y	Y
5. Were the outcomes assessed using objective criteria?	N	Y	Y	Y	Y	Y
6. Was follow-up carried out over a sufficient time period?	Y	Y	Y	Y	Y	Y
7. Were the outcomes of people who withdrew described and included in the analysis?	N	Y	Y	Y	Y	Y
8. Were outcomes measured in a reliable way?	N	Y	U	Y	Y	Y
9. Was appropriate statistical analysis used?	Y	Y	Y	Y	Y	Y
% yes/risk	6.6% Moderate	00%Low	00% Low	00% Low	00% Low	8.8% Low

Caption: Y = Yes; N = No; U = Unclear; NA = Not applicable
